# Supplementary material for: Applying Physiologically Based Pharmacokinetic Modeling to Interpret Carbamazepine’s Nonlinear Pharmacokinetics and Its Induction Potential on Cytochrome P450 3A4 and Cytochrome P450 2C9 Enzymes
Source: Pharmaceutics. 2024 May 30;16(6):737. doi: 10.3390/pharmaceutics16060737 (PMC11206836; doi:10.3390/pharmaceutics16060737)
Supplement: Supplementary file 1 [file pharmaceutics-16-00737-s001.zip › pharmaceutics-3022741-supplementary.pdf]

pharmaceutics

Applying Physiologically Based Pharmacokinetic Modeling to Interpret Carbamazepine Nonlinear Pharmacokinetics and its induction Potential on CYP3A4 and CYP2C9 Enzymes

Supplementary Materials

Xuefen Yin [1], Brian Cicali [1], Leyanis Rodriguez-Vera [1], Viera Lukacova[2], Rodrigo Cristofoletti [1*], and Stephan Schmidt [1*]

[1] Center for Pharmacometrics and Systems Pharmacology, Department of Pharmaceutics, College of Pharmacy, University of Florida, Orlando, FL, USA.
[2] Simulations Plus, Lancaster, CA, USA; viera.lukacova@simulations-plus.com (V.L.);

Funding: This research was funded by Simulations-Plus, funding ID: AGR00020791.

Conflict of Interest: All authors declared no conflicts of interest directly relevant to the content of this manuscript.

Corresponding Authors

Rodrigo Cristofoletti, Ph.D. & Stephan Schmidt, Ph.D.
Center for Pharmacometrics and Systems Pharmacology
Department of Pharmaceutics, College of Pharmacy,
University of Florida,
6550 Sanger RD. Orlando, FL. 32827. US
Phone: +1-407-313-7050 and +1 407 313 7012
Email: rcristofoletti@ufl.edu and sschmidt@cop.ufl.edu

Tables

Table S1 CBZ-E PBPK Model performance- Simulated versus observed PK parameters of CBZ-E oral single doses in healthy subjects. 4

Table S2 The AAFE of predicted CBZ-E plasma concentrations for CBZ-E administration 4

Table S3 CBZ P-M PBPK Model performance- Simulated versus observed PK parameters for CBZ iv and oral single doses in healthy subjects. 5

Table S4 The AAFE of predicted CBZ and CBZ-E concentrations for CBZ single dose administration 8

Table S5 CBZ P-M PBPK Model performance - Simulated versus observed PK parameters for CBZ oral multiple doses in subjects. 9

Table S6 Substrate model performance - Simulated versus observed AUClast and Cmax with and without co-administer CBZ. 10

Table S7 DDI application - Simulated versus observed DDI AUClast and Cmax ratios. 10

Table S8 The AAFE of predicted CBZ plasma concentrations for CBZ multiple dose administration 11

Table S9 The clinical observed data for CBZ nonlinear PK exploration....11

Table S10 The clinical simulation results for CBZ nonlinear PK exploration 12

Table S11 Summary of induction parameters of CBZ on enzyme CYP3A4....13

Figures

Figure S1 Predicted compared to observed CBZ-E plasma concentration-time profiles (linear) after oral administration of CBZ-E. 14

Figure S2 Predicted compared to observed CBZ-E plasma concentration-time profiles(Semi-logarithmic) after oral administration of CBZ-E. 16

Figure S3 goodness of fit plots for CBZ-E PBPK model performance. 18

Figure S4 Predicted compared to observed CBZ and CBZ-E plasma concentration-time profiles (linear) after intravenous and oral administration of CBZ. 19

Figure S5 Predicted compared to observed CBZ and CBZ-E plasma concentration-time profiles (Semi-logarithmic) after intravenous and oral administration of CBZ. 27

Figure S6 Model predictions comparison of CBZ concentration-time profiles in fast and fed conditions....34

Figure S7 Predicted compared to observed CBZ plasma concentration-time profiles(linear) after oral administration of CBZ....35

Figure S8 Predicted compared to observed CBZ plasma concentration-time profiles (Semi-logarithmic) after oral administration of CBZ....37

Figure S9 Summary plot for all clinical observed data when given 200mg CBZ IR tablet. 39

Figure S10 Summary plot for all clinical observed data when given 400mg CBZ IR tablet. 39

Figure S11 Summary plot for all clinical observed data when given 600mg CBZ IR tablet. 39

Figure S12 CBZ induction capacity for different enzymes. 40

Figure S13 CBZ nonlinear PK exploration for single dose. 40

Figure S14 CBZ-E PBPK model sensitivity analysis. 41

Figure S15 CBZ P-M PBPK model sensitivity analysis calculated as change of the simulated CBZ $AUC_{0\text{-}inf}$. 41

Figure S16 CBZ P-M PBPK model sensitivity analysis calculated as change of the simulated CBZ-E $AUC_{0\text{-}inf}$. 42

Figure S17 Comparing Simulated and Predicted Phase I Results of Phenytoin 42

Figure S18 Comparative Analysis of Observed Phenytoin DDI Study Values 43

Figure S19 The clinical simulation results for CBZ nonlinear PK exploration 444

Table S1 CBZ-E PBPK Model performance- Simulated versus observed PK parameters of CBZ-E oral single doses in healthy subjects.

Dose (mg)	Route	Data set	Gender (name)	Age (years)	Weight (kg)	Healthy (%)	AUC_{0-inf} (µg-h/mL)			AUC_{0-t} (µg-h/mL)			C_{max} (µg/mL)			Reference
							Pre	Obs	Pre/Obs	Pre	Obs	Pre/Obs	Pre	Obs	Pre/Obs	
50	sus, sd	test	1M(TT)	31	65	100	9.604	12.36	0.78	9.248	11.41	0.81	1.149	1.24	0.93	Tomson 1983[1]
50	sus, sd	test	1M(TV)	27	63	100	9.597	11.06	0.87	9.276	10.58	0.88	1.18	1.043	1.13	Tomson 1983[1]
100	sus, sd	test	1M(TT)	31	65	100	19.21	16.1	1.19	18.5	15.35	1.21	2.298	1.476	1.56	Tomson 1983[1]
100	sus, sd	training	1M(CT)	34	69	100	19.23	18.57	1.04	18.37	17.9	1.03	2.182	1.9	1.15	Tomson 1983[1]
100	sus, sd	test	1F(GB)	50	53	100	19.07	13.77	1.38	18.74	13.18	1.42	2.864	1.695	1.69	Tomson 1983[1]
150 (121.5)	sol, sd	test	4M 2F	25.7[23-32]	56.2[44-78]	-	23.26	20.57	1.13	22.74	20.1	1.13	3.108	2.35	1.32	Sumi 1987[2]
200	sus, sd	test	1M(CT)	34	69	100	38.47	37.37	1.03	36.75	36.24	1.01	4.366	3.596	1.21	Tomson 1983[1]
200	sus, sd	test	1F(GB)	50	53	100	38.13	40.6	0.94	37.48	38.15	0.98	5.74	4.219	1.36	Tomson 1983[1]
100	e-tab, sd	training	4M 2F	[20-43]	[55-75]	100	16.13	17.5	0.92	15.32	16.88	0.91	1.082	1.078	1.00	Pisani 1990[3]
100	e-tab, sd	test	4M 2F	[20-50]	[50-74]	100	16.13	11.69	1.38	15.32	11.02	1.39	1.082	0.95	1.14	Pisani 1992[4]

e-tab: enteric-coated tablet. sus: suspension; sol: solution; Pre: predicted value; Obs: observed value. A PBPK model representing the average subject (gender/age/weight) in a given clinical study was used in each simulation. If mean age and/or body is not mentioned, 30 years and 75 Kg will be the default values. The color boxes are shown the PK parameters ratio (simulated versus observed): the green boxes mean the values were within 1.25-fold error, yellow were within 2-fold deviation and red were outside 2-fold error. Simulation integration timestep:0.1h.

Table S2 The AAFE of predicted CBZ-E plasma concentrations for CBZ-E administration

**Dose (mg)**	**Route**	**Gender(name)**	**AAFE**	**Reference**
50	sus, sd	1M(TT)	1.30	Tomson 1983[1]
50	sus, sd	1M(TV)	1.18	Tomson 1983[1]
100	sus, sd	1M(TT)	1.18	Tomson 1983[1]
100	sus, sd	1M(CT)	1.16	Tomson 1983[1]
100	sus, sd	1F(GB)	1.35	Tomson 1983[1]
150	sol, sd	4M 2F	1.18	Sumi 1987[2]
200	sus, sd	1M(CT)	1.19	Tomson 1983[1]
200	sus, sd	1F(GB)	1.13	Tomson 1983[1]
100	e-tab, sd	4M 2F	1.20	Pisani 1990[3]
100	e-tab, sd	4M 2F	1.34	Pisani 1992[4]
**Mean AAFE (range)**			**1.22(1.13-1.35)**	
			**10/10 with AAFE < 2**	

e-tab: enteric-coated tablet. sus: suspension; sol: solution;

Table S3 CBZ P-M PBPK Model performance- Simulated versus observed PK parameters for CBZ iv and oral single doses in healthy subjects.

Dose (mg)	Route	Data set	Gender (name)	Age (years)	Weight (kg)	Healthy (%)	CBZ-E measure	$AUC_{0\text{-}inf}$ (μg-h/mL)			$AUC_{0\text{-}t}$ (μg-h/mL)			C_{max} (μg/mL)			Reference
								Pre	Obs	Pre/Obs	Pre	Obs	Pre/Obs	Pre	Obs	Pre/Obs	
**Carbamazepine (Parent drug)**																	
10	iv(2h)	training	1M (S1)	43	76	100	No	7.446	10.54	0.71	6.995	9.847	0.71	0.17	0.14	1.21	Gerardin 1990[5]
10	iv(2h)	test	1M (S2)	49	89	100	No	7.167	11.45	0.63	6.530	10.3	0.63	0.15	0.15	0.10	Gerardin 1990[5]
50	sol	test	1M	29	-	100	No	36.45	31.54	1.16	26.43	25.58	1.03	0.80	0.59	1.37	Rawlins 1975[6]
100	sus	training	1M (S1)	43	76	100	No	72.96	80.76	0.90	68.54	75.93	0.90	1.44	1.45	0.99	Gerardin 1990[5]
100	sus	test	1M (S2)	49	89	100	No	70.23	93.42	0.75	63.97	81.16	0.79	1.23	1.81	0.68	Gerardin 1990[5]
100	sol	test	1M	29	-	100	No	72.98	49.61	1.47	59.09	45.24	1.31	1.57	1.33	1.19	Rawlins 1975[6]
200	sus	test	1F	29	62	100	No	150.7	194.2	0.78	124.5	166.5	0.75	2.96	4.00	0.74	Eichelbaum 1985[7]
200	Sol	test	1M	29	-	100	No	146.2	148.7	0.98	117.8	122.6	0.96	2.95	3.01	0.98	Rawlins 1975[6]
200	Sol	test	4M 2F	25.7[23-32]	56.2[44-78]	-	No	156.9	238.2	0.66	103.9	136.3	0.76	4.08	4.56	0.90	Sumi 1987[2]
200	Sus	test	1F(GB)	50	53	100	No	150.1	131.2	1.14	124.8	123.5	1.01	3.12	4.36	0.72	Tomson 1983[1]
200	sus	test	9M	-	69.1[53-104]	100	No	149.4	160.1	0.93	86.65	104.3	0.83	3.06	3.49	0.88	Wada 1978[8]
100	tab	test	6M	-	75[69-84]	100	No	72.31	59.43	1.22	67.97	58.82	1.16	1.11	0.94	1.18	Gerardin 1976 [9]
200	tab	test	12M	29.2[25-30]	67.5[55-81]	100	Yes	146.6	192.7	0.76	121.2	172.5	0.70	2.40	3.33	0.72	Bedada 2015[10]
200	tab	test	12M	[28-32]	[58-76]	100	Yes	146.8	194	0.76	121.7	168.2	0.72	2.42	3.13	0.78	Bedada 2017[11]
200	tab	test	1M	29	-	100	No	143.2	123.6	1.16	134.2	116.9	1.15	2.12	1.84	1.16	Rawlins 1975[6]
200	tab	test	6M	-	75[69-84]	100	No	143.6	114.6	1.25	134.9	110.9	1.22	2.15	1.64	1.31	Gerardin 1976[9]
200	tab	test	10M	[22-35]	[62-75]	100	Yes	146.2	297.6	0.49	129.5	264.8	0.49	2.36	3.64	0.65	Kim 2005[12]
200	tab	training	24M	[21-35]	[61-93]	100	No	142.8	143.1	1.00	133.7	134.9	0.99	2.09	1.77	1.18	Meyer 1992[13]
200	tab	test	16M 4F	[22-36]	[50-98]	100	No	142.8	171.3	0.83	133.7	78.59	1.70	2.09	1.90	1.11	Meyer 1998[14]
200	tab	test	8M	23[20-25]	70[65-75]	100	No	145.7	420.4	0.35	104.2	360.7	0.29	2.31	10.17	**0.23**	Shahzadi 2011[15]
200	tab	test	12M	[22-35]	-	100	No	143.6	164.4	0.87	124	147	0.84	2.15	2.58	0.84	Saint-Salvi1987 [16]
200	tab	test	9M	-	69.1[53-104]	100	No	145.9	176.8	0.83	119.7	96.36	1.24	2.34	2.47	0.95	Wada 1978[8]
400	tab, fed	test	7M	[21-27]	[70-90]	100	Yes	285.5	363.4	0.79	221.2	280	0.79	4.03	5.10	0.79	Barzaghi 1987[17]
400	tab	test	24M	[20-40]	-	100	Yes	279.6	305.3	0.92	192.2	227.9	0.84	4.03	4.82	0.84	Bianchetti1987[18]

400	tab	test	1M(S1)	37	78	100	Yes	278.5	250.7	1.11	217.1	217.9	1.00	3.89	3.36	1.16	Faigle 1975[19]
400	tab	test	1M(S2)	34	83	100	Yes	274.2	250.4	1.10	208.5	203.8	1.02	3.65	3.35	1.09	Faigle 1975[19]
400	tab	test	18M	[29-37]	72[70-81]	100	No	281.2	226.3	1.24	245.1	221.5	1.11	4.18	3.76	1.12	Kovacevic 2009[20]
400	tab	test	5M	25[24-27]	72[62-82]	100	No	281.4	242.4	1.16	153	167.8	0.91	4.18	4.91	0.85	Morselli 1975[21]
400	tab	test	6M	[21-22]	[62-77]	100	No	282.6	297.9	0.95	230.1	263.6	0.87	4.31	4.92	0.88	Pynnonen 1977 [22]
400	tab	test	8M	[24-36]	[72.3-96.4]	100	No	273.4	365.4	0.75	177.5	231.2	0.77	3.60	4.33	0.83	Wong 1983[23]
415.8	tab	test	3M 3F	25.8	69.3	100	No	292.8	297.2	0.99	208.9	213.1	0.98	4.47	4.52	0.99	Levy 1975[24]
415.8	tab, fed	test	3M 3F	25.8	69.3	100	No	305.9	290	1.05	221.4	229.7	0.96	4.85	5.51	0.88	Levy 1975[24]
600	tab	test	8M	[24-35]	83.4	100	Yes	394.9	215.6	1.83	355.2	200.9	1.77	5.05	3.53	1.43	Dalton 1985[25]
600	tab	test	8M	[23-26]	81	100	Yes	398.1	333.4	1.19	361	316.6	1.14	5.20	4.50	1.16	Dalton 1985(a)[26]
600	tab	test	6M	-	75[69-84]	100	No	401.9	296.6	1.36	376.7	289.9	1.30	5.56	4.41	1.26	Gerardin 1976[9]
800	tab	test	3M 3F	[21-32]	-	100	No	507.7	534.9	0.95	435	506.1	0.86	6.74	9.70	0.70	Cotter 1977[27]
400	CR-tab	test	-	-	-	-	No	200.9	218.5	0.92	172.3	179.8	0.96	2.62	2.63	1.00	Graf 1990[28]
400	CR-tab	test	34M	[20-55]	-	100	Yes	200.9	255.7	0.79	188.2	233.7	0.81	2.62	2.27	1.16	Licht 2005[29]
400	CR-tab	training	24-	31.8[20-52]	69.8[50-96]	100	No	202.3	239.7	0.84	186.1	225.3	0.83	2.81	3.20	0.88	Kovacevic 2009[30]
400	CR-tab, fed	training	13M	[18-45]	-	100	No	277.3	356.4	0.78	272.3	338.8	0.80	4.05	4.07	1.00	Kshirssagar 2014[31]
600	CR-tab	test	19M	24[19-27]	75	100	No	301.6	308.5	0.98	272.8	286	0.95	3.93	3.54	1.11	Licht 2005[29]
300	CR-cap	test	8M 4F	-	-	-	Yes	181.1	175.9	1.03	177.7	174.2	1.02	2.41	1.83	1.32	Gande 2009[32]
400	CR-cap	training	12M	33.8[21-48]	81.3	100	Yes	238.1	232.3	1.02	229.3	229.2	1.00	2.98	3.06	0.97	McLean 2001[33]
400	CR-cap, fed	training	12M	33.8[21-48]	81.3	100	Yes	283.9	263.1	1.08	273.8	259.7	1.05	4.04	4.10	0.99	McLean 2001[33]
**Carbamazepine-10,11-epoxide (active metabolite)**																	
200	tab	test	12M	29.2[25-30]	67.5[55-81]	100	Yes	15.08	13.61	1.11	11.83	10.81	1.09	0.19	0.16	1.13	Bedada 2015[10]
200	tab	test	12M	[28-32]	[58-76]	100	Yes	15.04	14.57	1.03	11.84	11.37	1.04	0.19	0.18	1.06	Bedada 2017[11]
200	tab	test	10M	[22-35]	[62-75]	100	Yes	15.14	34.33	0.44	13.00	30.41	0.43	0.18	0.36	0.51	Kim 2005[12]
400	tab, fed	test	7M	[21-27]	[70-90]	100	Yes	32.14	16.84	1.91	23.07	12.69	1.82	0.33	0.19	1.73	Barzaghi 1987[17]
400	tab	test	24M	[20-40]	-	100	Yes	30.78	17.35	1.77	18.55	10.6	1.75	0.33	0.21	1.59	Bianchetti1987[18]
400	tab	test	1M(S1)	37	78	100	Yes	30.90	33.46	0.92	22.37	28.49	0.79	0.33	0.40	0.82	Faigle 1975[19]
400	tab	test	1M(S2)	34	83	100	Yes	30.90	29.65	1.04	22.37	27.13	0.82	0.33	0.44	0.75	Faigle 1975[19]

600	tab	test	8M	[24-35]	83.4	100	Yes	45.20	16.98	2.66	39.52	16.08	2.46	0.45	0.28	1.62	Dalton 1985[25]
600	tab	test	8M	[23-26]	81	100	Yes	44.81	19.78	2.27	39.59	19.48	2.03	0.46	0.27	1.68	Dalton 1985(a)[26]
400	CR-tab	test	34M	[20-55]	-	100	Yes	21.78	12.36	1.76	20.07	12.06	1.66	0.24	0.12	1.98	Licht 2005[29]
300	CR-cap	test	8M 4F	-	-	-	Yes	19.64	9.06	2.17	19.18	9.08	2.11	0.21	0.08	2.66	Gande 2009[32]
400	CR-cap	training	12M	33.8[21-48]	81.3	100	Yes	27.82	11.93	2.33	15.13	8.87	1.71	0.27	0.18	1.51	McLean 2001[33]
400	CR-cap, fed	training	12M	33.8[21-48]	81.3	100	Yes	32.93	16.34	2.02	18.56	12.98	1.43	0.33	0.24	1.38	McLean 2001[33]

e-tab: enteric-coated tablet. sus: suspension; sol: solution; tab: immediate release tablet; CR-tab: control/extended/sustained release tablet; CR-cap: control/extended/sustained release capsule; Pre: predicted value; Obs: observed value. A PBPK model representing the average subject (gender/age/weight) in a given clinical study was used in each simulation. If mean age and/or body is not mentioned, 30 years and 75 Kg will be the default values. The color boxes are shown the PK parameters ratio (simulated versus observed): the green boxes mean the values were within 1.25-fold error, yellow were within 2-fold deviation and red were outside 2-fold error. Water consumption(ml): 240 for Meyer 1998, Gerardin 1976, Bedada 2015; 180 for Dalton 1985; 150 for Bianchetti 1987. Simulation integration timestep:0.1h.

Table S4 The AAFE of predicted CBZ and CBZ-E concentrations for CBZ single dose administration

Dose (mg)	Route	Gender(name)	AAFE		Reference
			CBZ	CBZ-E	
10	iv(2h)	1M (S1)	**1.28**	-	Gerardin 1990[5]
10	iv(2h)	1M (S2)	1.29	-	Gerardin 1990[5]
50	sol	1M	1.17	-	Rawlins 1975[6]
100	sus	1M (S1)	1.21	-	Gerardin 1990[5]
100	sus	1M (S2)	1.29	-	Gerardin 1990[5]
100	sol	1M	1.32	-	Rawlins 1975[6]
200	sus	1F	1.27	-	Eichelbaum 1985[7]
200	sol	1M	1.15	-	Rawlins 1975[6]
200	sol	4M 2F	1.31	-	Sumi 1987[2]
200	sus	1F(GB)	1.68	-	Tomson 1983[1]
200	sus	9M	1.27	-	Wada 1978[8]
100	tab	6M	1.41	-	Gerardin 1976[9]
200	tab	12M	1.37	1.18	Bedada 2015[10]
200	tab	12M	1.27	1.25	Bedada 2017[11]
200	tab	1M	1.20	-	Rawlins 1975[6]
200	tab	6M	1.35	-	Gerardin 1976[9]
200	tab	10M	1.69	2.74	Kim 2005[12]
200	tab	24M	1.18	-	Meyer 1992[13]
200	tab	16M 4F	1.22	-	Meyer 1998[14]
200	tab	8M	3.67	-	Shahzadi 2011[15]
200	tab	12M	1.19	-	Saint-Salvi1987 [16]
200	tab	9M	1.22	-	Wada 1978[8]
400	tab, fed	7M	1.28	1.70	Barzaghi 1987[17]
400	tab	24M	1.31	1.55	Bianchetti1987[18]
400	tab	1M(S1)	1.23	1.51	Faigle 1975[19]
400	tab	1M(S2)	1.08	1.40	Faigle 1975[19]
400	tab	18M	1.20	-	Kovacevic 2009[20]
400	tab	5M	1.16	-	Morselli 1975[21]
400	tab	6M	1.13	-	Pynnonen 1977 [22]
400	tab	8M	1.29	-	Wong 1983[23]
415.8	tab	3M 3F	1.09	-	Levy 1975[24]
415.8	tab, fed	3M 3F	1.11	-	Levy 1975[24]
600	tab	8M	1.89	2.18	Dalton 1985[25]
600	tab	8M	1.42	2.28	Dalton 1985(a)[26]
600	tab	6M	1.42	-	Gerardin 1976[9]
800	tab	3M 3F	1.31	-	Cotter 1977[27]
400	CR-tab	-	1.14	-	Graf 1990[28]
400	CR-tab	34M	1.51	2.37	Licht 2005[29]
400	CR-tab	24-	1.17	-	Kovacevic 2009[30]
400	CR-tab, fed	13M	1.45	-	Kshirssagar 2014[31]
600	CR-tab	19M	1.23	-	Licht 2005[29]
300	CR-cap	8M 4F	1.30	2.64	Gande 2009[32]
400	CR-cap	12M	1.17	1.76	McLean 2001[33]
400	CR-cap, fed	12M	1.54	1.49	McLean 2001[33]
**Mean AAFE (range)**			**1.35(1.08-3.67)**	**1.85 (1.18-2.74)**	
			**43/44 with AAFE < 2**	**8/13 with AAFE < 2**	

e-tab: enteric-coated tablet. sus: suspension; sol: solution; tab: immediate release tablet; CR-tab: control/extended/sustained release tablet; CR-cap: control/extended/sustained release capsule.

Table S5 CBZ P-M PBPK Model performance - Simulated versus observed PK parameters for CBZ oral multiple doses in subjects.

Dose (mg)	Route	Data set	Gender (name)	Age (years)	Weight (kg)	Healthy (%)	AUC_{last} (μg-h/mL)			C_{max} (μg/mL)			Reference
							Pre	Obs	Pre/Obs	Pre	Obs	Pre/Obs	
100(D1-D3)/ 200(D4-D6)/ 400(D7-D21)	tab, bid tab, bid tab, qd	test	5M 3F	24-43	-	100	157.0	142.9	1.10	7.39	7.07	1.05	Burstein 2000[34]
100(D1-D3)/ 200(D4-D6)/ 400(D7-D21)	tab, bid tab, bid tab, qd	test	16M	18-45	-	100	157.0	137.7	1.14	7.47	7.16	1.04	Moller 2001[35]
200(D1-D3)/ 200(D4-D6)/ 400(D7-D21)	tab, qd tab, bid tab, qd	training	25M 11F	30 (20-45)	75.5 (64-92)	100	157.1	141.7	1.11	7.45	7.44	1.00	Ji 2008[36]
357(D1-D14)	tab, qd	test	7M	25 (22-27)	78.8 (70.8-90.9)	100	143.0	151.1	0.95	6.80	7.12	0.95	Miles 1989[37]
600(D1) 600(D5)	tab, sd tab, sd	test	6M	23.3 (22-26)	67.5 (62-76)	100	296.7	356.2	0.83	5.64	5.61	1.00	Bernus 1994[38]
200(D1) 200(D5-D17)	tab, qd tab, qd	test	5M	-	77.4 (69-84)	100	207.4	156.9	1.32	4.44	3.51	1.27	Gerardin 1976[9]
200(D1) 200(D5-D17)	tab, qd tab, qd	test	1M(S1)	-	84	100	207.4	130.9	1.58	4.44	3.60	1.23	Gerardin 1976[9]
200(D1) 200(D5-D17)	tab, qd tab, qd	test	1M(S2)	-	82	100	202.6	189.6	1.07	4.44	3.84	1.16	Gerardin 1976[9]
200(D1) 200(D5-D17)	tab, qd tab, qd	test	1M(S3)	-	73	100	207.4	189.3	1.10	4.44	4.26	1.04	Gerardin 1976[9]
200(D1) 200(D5-D17)	tab, qd tab, qd	test	1M(S4)	-	69	100	207.4	135.4	1.53	4.44	3.44	1.29	Gerardin 1976[9]
200(D1) 200(D5-D17)	tab, qd tab, qd	test	1M(S5)	-	79	100	207.4	140.6	1.48	4.44	2.94	1.51	Gerardin 1976[9]

tab: immediate release tablet; sd: single dose; qd: once daily; bid: twice times daily; tid: three times daily; Pre: predicted value; Obs: observed value; D: day. A PBPK model representing the average subject (gender/age/weight) in a given clinical study was used in each simulation. If mean age and/or body is not mentioned, 30 years and 75 Kg will be the default values. The color boxes are shown the PK parameters ratio (simulated versus observed): the green boxes mean the values were within 1.25-fold error, yellow were within 2-fold deviation and red were outside 2-fold error. Simulation integration timestep:0.1h.

Table S6 Substrate model performance - Simulated versus observed AUClast and Cmax with and without co-administer CBZ.

Perpetrator	Victim			Baseline						DDI						Reference
				AUC_{last} (μg-h/mL)			C_{max} (μg/mL)			AUC_{last} (μg-h/mL)			C_{max} (μg/mL)			
CBZ(tab) Dose regimen	Enzyme	Name	Dose regimen	Pre	Obs	Pre/Obs	Pre	Obs	Pre/Obs	Pre	Obs	Pre/Obs	Pre	Obs	Pre/Obs	
200 mg(D1-D3, bid); 400mg(D4-D17, bid)	CYP3A4	Quinidine	200mg, sd, cap	7.76	6.42	1.21	0.99	0.89	1.11	1.84	2.68	0.69	0.44	0.45	0.98	Andreasen 2007[39]
300mg(D1-D22, bid)	CYP3A4	Dolutegravir	50mg (D17-D22, tab)	67.91	55.80	1.22	4.73	3.75	1.26	34.63	29.11	1.19	3.19	2.47	1.29	Song 2016[40]
200mg(D1-D7,tid); 200mg, sd at D8	CYP2C9	Phenytoin	600mg,sd,cap	330	237	1.39	7.54	4.79	1.57	258	172.5	1.50	7.23	4.57	1.58	Lai 1992[41]

tab: immediate release tablet; cap: capsule; sd: single dose; qd: once daily; bid: twice times daily; tid: three times daily. Pre: predicted value; Obs: observed value**;** D: day. The color boxes are shown the PK parameters ratio (simulated versus observed): the green boxes mean the values were within 1.25-fold error, yellow were within 2-fold deviation and red were outside 2-fold error.

Table S7 DDI application - Simulated versus observed DDI AUClast and Cmax ratios.

Perpetrator	Victim			Demographics				DDI AUC_{last} ratio			DDI C_{max} ratio			Reference
CBZ(tab) Dose regimen	Enzyme	Name	Dose regimen	Gender	Age (years)	Weight (kg)	Healthy (%)	Pre	Obs	Pre/Obs	Pre	Obs	Pre/Obs	
200 mg(D1-D3, bid); 400mg(D4-D17, bid)	CYP3A4	Quinidine	200mg, sd, cap	10M	21-26	62-85	100	0.238	0.417	0.57	0.441	0.500	0.88	Andreasen 2007[39]
300mg(D1-D22, bid)	CYP3A4	Dolutegravir	50mg (D17-D22, tab)	14M 2F	39.6 (18-65)	82.4	100	0.510	0.522	0.98	0.675	0.660	1.02	Song 2016[40]
100mg(D1-D3,bid) 200mg(D4-D6,bid) 300mg(D7-D18,bid)	CYP3A4	Midazolam	2mg, sd, tab	-	-	-	100	0.103	0.211	0.49	0.183	0.318	0.58	Lutz 2018[41]
200mg(D1-D7,tid); 200mg, sd at D8	CYP2C9	Phenytoin	600mg,sd,cap	11M	20-25	62	100	0.782	0.728	1.07	0.959	0.953	1.01	Lai 1992[41]
100mg(D1-D3,bid) 200mg(D4-D6,bid) 300mg(D7-D18,bid)	CYP2C9	Tolbutamide	500mg, sd, tab	-	-	-	100	0.72	0.639	1.13	0.937	0.935	1.00	Lutz 2018[42]

tab: immediate release tablet; cap: capsule; sd: single dose; qd: once daily; bid: twice times daily; tid: three times daily; Pre: predicted value; Obs: observed value; D: day. The color boxes are shown the PK parameters ratio (simulated versus observed): the green boxes mean the values were within 1.25-fold error, yellow were within 2-fold deviation and red were outside 2-fold error.

Table S8 The AAFE of predicted CBZ plasma concentrations for CBZ multiple dose administration

Dose (mg)	Gender(name)	AAFE	Reference
100(D1-D3, bid)/200(D4-D6, bid)/400(D7-D21, qd)	5M 3F	1.12	Burstein 2000[34]
100(D1-D3, bid)/200(D4-D6, bid)/400(D7-D21, qd)	16M	1.12	Moller 2001[35]
200(D1-D3, qd)/200(D4-D6, bid)/400(D7-D21, qd)	25M 11F	1.12	Ji 2008[36]
357(D1 -D14, qd)	7M	1.06	Miles 1989[37]
600(D1, sd; D5, sd)	6M	1.20	Bernus 1994[38]
200(D1, qd; D5-D17, qd)	5M	1.20	Gerardin 1976[9]
200(D1, qd; D5-D17, qd)	1M(S1)	1.38	Gerardin 1976[9]
200(D1, qd; D5-D17, qd)	1M(S2)	1.12	Gerardin 1976[9]
200(D1, qd; D5-D17, qd)	1M(S3)	1.14	Gerardin 1976[9]
200(D1, qd; D5-D17, qd)	1M(S4)	1.34	Gerardin 1976[9]
200(D1, qd; D5-D17, qd)	1M(S5)	1.27	Gerardin 1976[9]
**Mean AAFE (range)**		**1.19(1.06-1.38)**	
		**11/11 with AAFE < 2**	

sd: single dose; qd: once daily; bid: twice times daily; tid: three times daily. Pre: predicted value; Obs: observed value; D: day.

Table S9 The clinical observed data for CBZ nonlinear PK exploration

Dose(mg)	$AUC_{0\text{-}inf}$ (μg/ml*hr)	$AUC_{0\text{-}inf}$/Dose (μg/ml*hr/mg)	n	literature
200	194	0.97	12	Bedada 2017[11]
200	123.6	0.62	1	Rawlins 1975 [6]
200	143.1	0.72	24	Meyer 1992 [43]
200	157.4	0.79	20	Meyer 1998[44]
200	114.9	0.57	6	Gerardin 1976[9]
200	176.8	0.88	9	Wada 1978 [8]
200	164.4	0.82	12	Saint-salvi 1987 [2]
200	192.7	0.96	12	Bedada 2015[10]
400	239.1	0.60	18	Kovacevic 2009[45]
400	297.9	0.74	6	Pynnonen 1977[22]
400	242.4	0.61	5	Morselli 1975[46]
400	305.3	0.76	24	Bianchetti 1987[18]
400	365.4	0.91	8	Wong 1983[23]
400	250.7	0.63	1	Faigle1975 [19]
600	215.6	0.36	8	Dalton 1985[25]
600	296.3	0.49	6	Gerardin 1976[9]
600	333.4	0.56	8	Dalton 1985(a)[26]

All studies are given a single-dose immediate-release tablet(IR tablet) and in fasted condition. n: subject size of the study. The values of $AUC_{0\text{-}inf}$ are calculated based on the digitized values from the literature using PKPlus in GastroPlus®.

Table S9 The clinical simulation results for CBZ nonlinear PK exploration

Dose (mg)	Single dose_IR tablet							Single dose_iv infusion		
	Dis%	Fa%	FDp%	F%	CL (L/h)	AUC_{0-inf} (μg*h/ml)	AUC_{0-inf}/Dose (μg*h/ml/mg)	CL (L/h)	AUC_{0-inf} (μg*h/ml)	AUC_{0-inf}/Dose (μg*h/ml/mg)
50	97.87	97.37	96.66	95.04	1.33	35.63	0.71	1.34	37.34	0.75
100	97.41	96.85	96.18	94.57	1.33	70.94	0.71	1.34	74.70	0.75
200	96.24	95.59	94.96	93.37	1.33	140.20	0.70	1.34	149.50	0.75
300	94.76	94.01	93.42	91.85	1.33	207.00	0.69	1.34	224.50	0.75
400	92.96	92.14	91.58	90.05	1.33	270.70	0.68	1.34	299.50	0.75
500	90.89	90.02	89.49	87.99	1.33	330.90	0.66	1.33	374.70	0.75
600	88.63	87.73	87.22	85.76	1.33	387.20	0.65	1.33	450.00	0.75
700	86.27	85.35	84.86	83.45	1.33	439.70	0.63	1.33	525.40	0.75
800	83.87	82.94	82.47	81.10	1.33	488.60	0.61	1.33	600.90	0.75
Dose (mg)	Multiple dose(qd)_IR tablet							Multiple dose(qd)_iv infusion		
	Dis%	Fa%	FDp%	F%	CL (L/h)	AUC_{24} (μg*h/ml)	AUC_{24}/Dose (μg*h/ml/mg)	CL (L/h)	AUC_{24} (μg*h/ml)	AUC_{24}/Dose (μg*h/ml/mg)
50	97.86	97.36	96.18	94.36	1.56	30.31	0.61	1.52	32.80	0.66
100	97.39	96.84	95.48	93.52	1.71	54.55	0.55	1.68	59.36	0.59
200	96.18	95.54	94.03	91.86	1.94	94.54	0.47	1.92	104.20	0.52
300	94.65	93.92	92.35	90.06	2.11	128.06	0.43	2.09	143.27	0.48
400	92.80	92.00	90.41	88.04	2.24	157.29	0.39	2.23	179.07	0.45
500	90.66	89.81	88.23	85.81	2.34	183.16	0.37	2.35	212.74	0.43
600	88.31	87.44	85.88	83.44	2.43	206.23	0.34	2.45	244.89	0.41
700	85.85	84.95	83.43	80.99	2.50	226.93	0.32	2.54	275.89	0.39
800	83.30	82.40	80.91	78.49	2.56	245.51	0.31	2.61	306.02	0.38

qd: once a day; CL: total systemic clearance; Dis%, Fa%, FDp% and F are represented the fraction of drug dissolved, in enterocytes, in portal vein, in systemic circulation, respectively.

Table S10 Summary of induction parameters of CBZ on enzyme CYP3A4

	Enzyme Activity Increase			mRNA Increase			Reference
Sample	Emax	EC50(μM)	Emax/EC50	Emax	EC50(μM)	Emax/EC50	
Donor 1	15.73	15.3	1.03	14.3	20.55	0.70	Shou 2008[47]
Donor 2	6.04	14.37	0.42	4.57	27.7	0.16	
Hu1624				7.8	10.5	0.74	Moore 2016[48]
BPB				4.1	10.5	0.39	
NON				10.7	14.2	0.75	
ACB	1.3	12	0.11	8.9	18	0.49	Savaryn 2022[49]
VJX	2.6	29	0.09	57	59	0.97	
Pooled				8.27	16	0.52	Zhang 2015[50]
CD-Hu4237				19	35	0.54	Vermet 2016[51]
BD-295				15	59	0.25	
IVT-IBG				9.3	36	0.26	
BD-281				13	98	0.13	
CD-Hu8084				19	95	0.20	
HH1024				21	29	0.72	
NON				11	31	0.35	Sun 2017[52]
Hu1624				10	23	0.43	
cdp				5.3	23	0.23	
Lot RCP				10.2	60	0.17	Fahmi 2008[53]
Lot Hu4026				34.3	55.8	0.61	
Donor 1				10.7	80.7	0.13	Kuramoto 2017[54]
Donor 2				11.9	47.5	0.25	
Donor 3				3.75	52.4	0.07	
Lot2B				16	108	0.15	Zhou 2017[55]
Lot3A				10	65	0.15	
Lot3B				14	53	0.26	
HH205	7.7	40	0.19	23	42	0.19	McGinnity 2009[56]
HH215	6.3			31			
Mean	6.67	22.13	0.37	14.31	44.99	0.38	
Max	15.73	40.00	1.03	57.00	108.00	0.97	
Min	1.30	12.00	0.09	3.75	10.50	0.07	

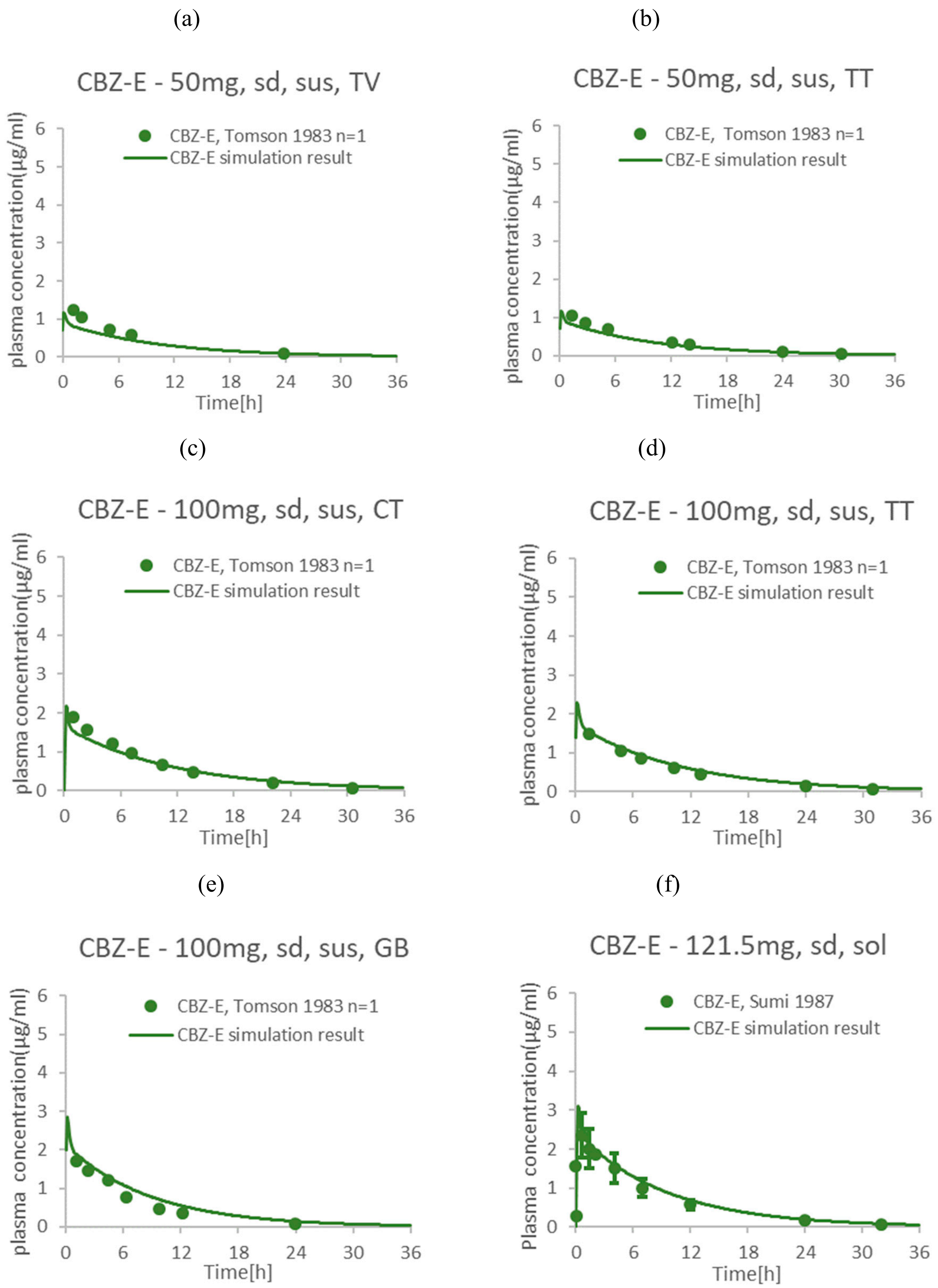

Figure S1 Predicted compared to observed CBZ-E plasma concentration-time profiles (linear) after oral administration of CBZ-E.
Observed data are shown as dots ± standard deviation; model predictions are shown as solid lines. sd: single dose; sol: solution; sus: suspension; tab: enteric-coated tablet.

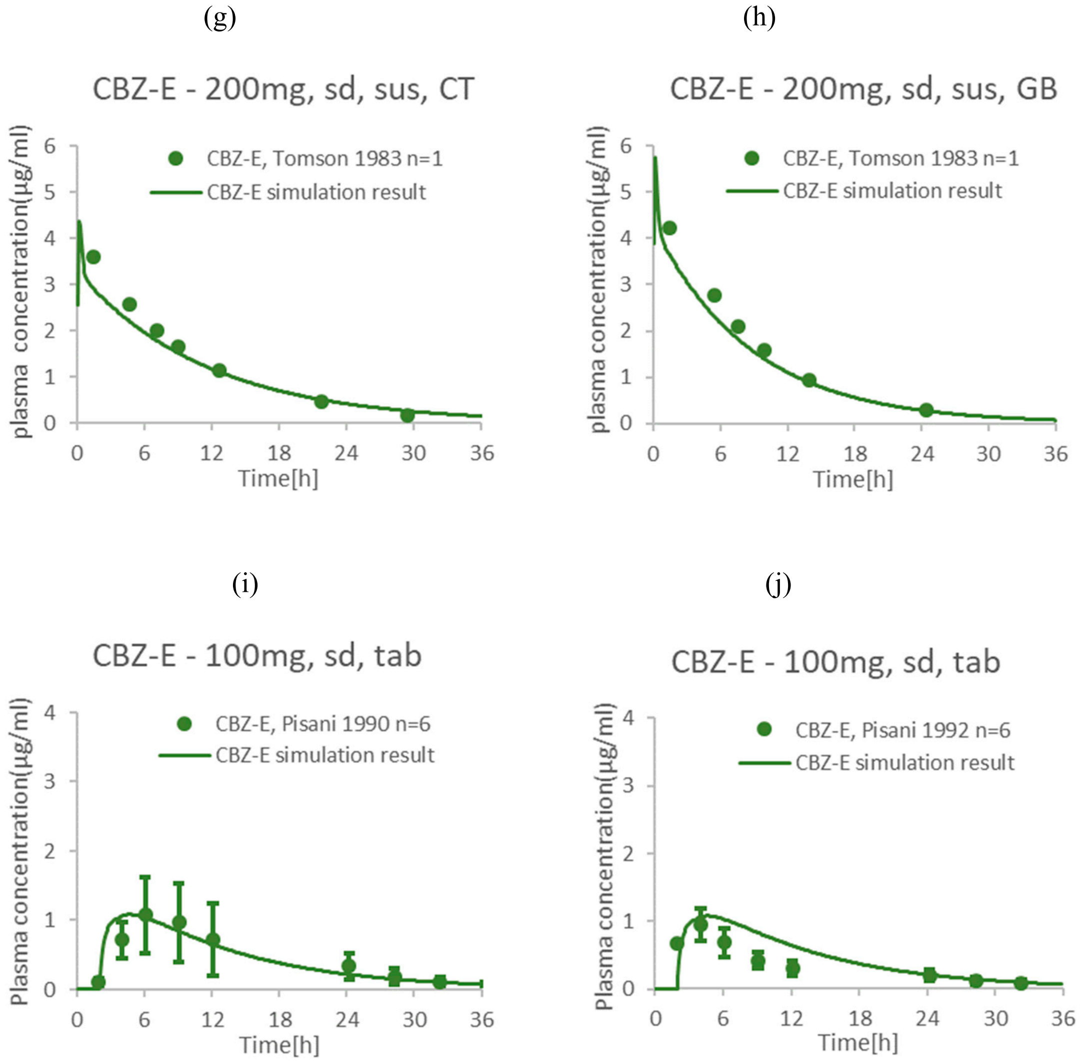

Figure S1 Predicted compared to observed CBZ-E plasma concentration-time profiles (linear) after oral administration of CBZ-E*(continued)*.

Observed data are shown as dots ± standard deviation; model predictions are shown as solid lines. sd: single dose; sol: solution; sus: suspension; tab: enteric-coated tablet.

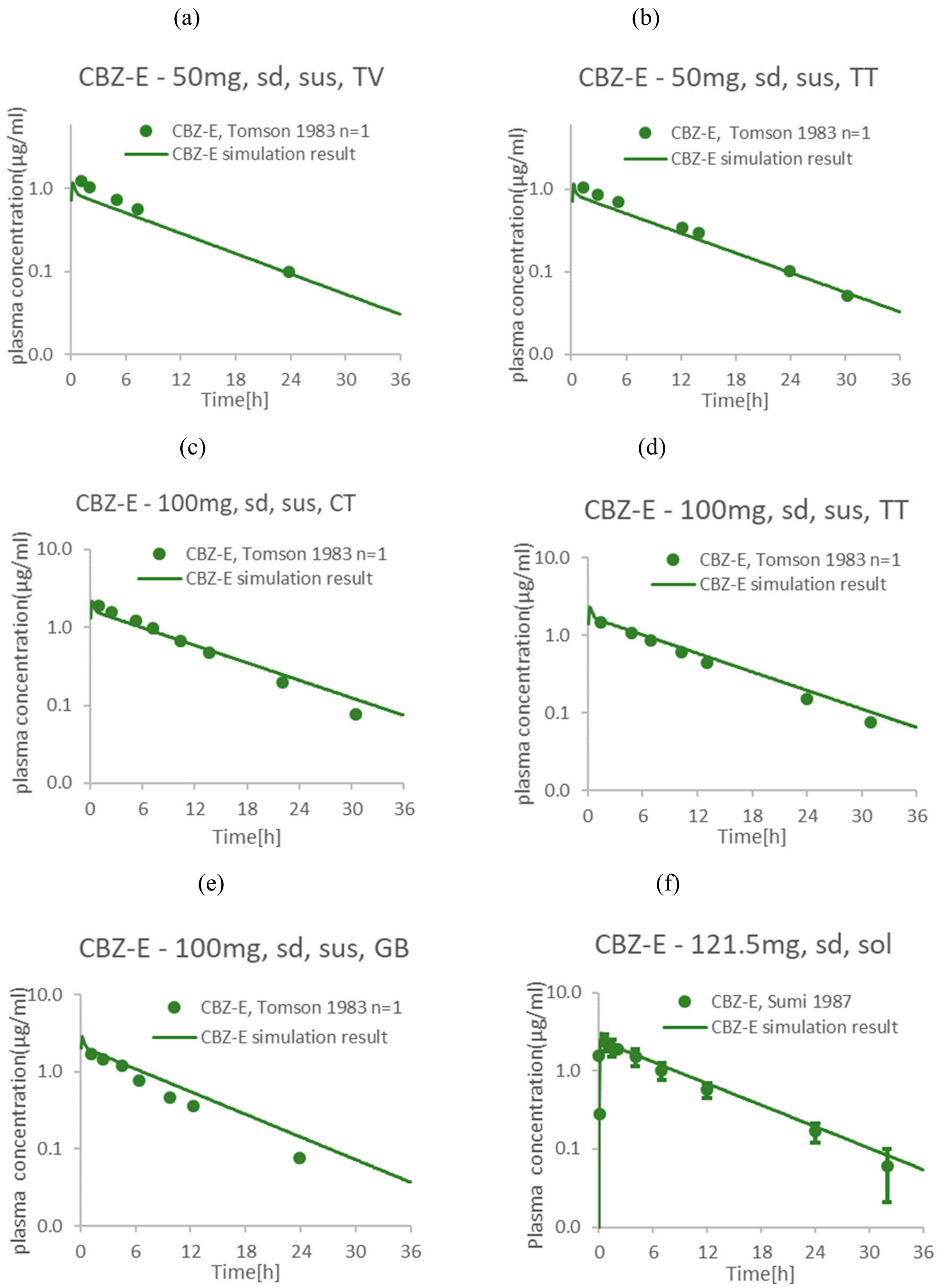

Figure S2 Predicted compared to observed CBZ-E plasma concentration-time profiles(Semi-logarithmic) after oral administration of CBZ-E.

Observed data are shown as dots ± standard deviation; model predictions are shown as solid lines. sd: single dose; sol: solution; sus: suspension; tab: enteric-coated tablet.

(g)

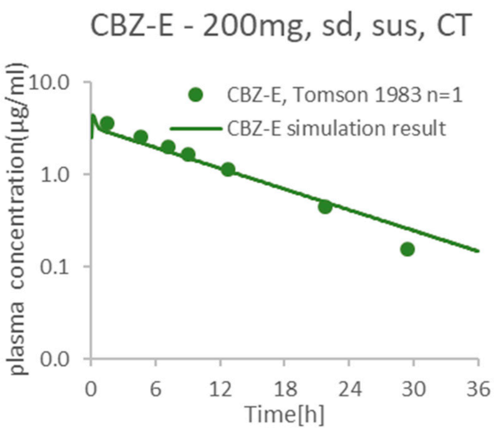

(h)

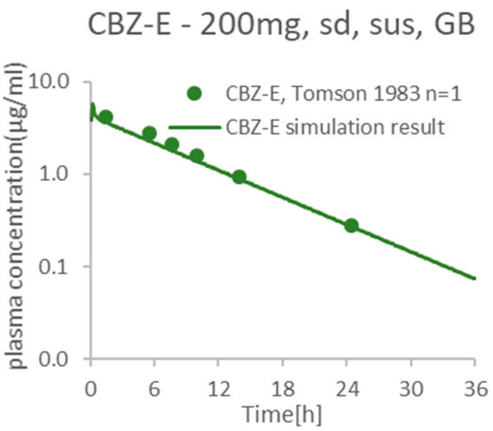

(i)

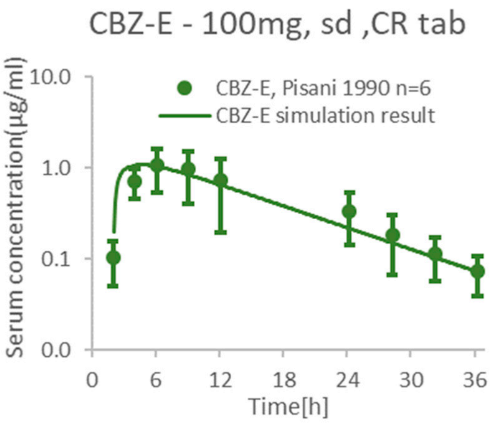

(j)

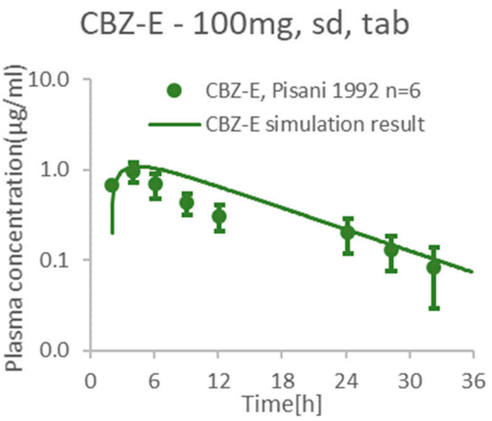

Figure S2 Predicted compared to observed CBZ-E plasma concentration-time profiles (Semi-logarithmic) after oral administration of CBZ-E *(continued).*

Observed data are shown as dots ± standard deviation; model predictions are shown as solid lines. sd: single dose; sol: solution; sus: suspension; tab: enteric-coated tablet.

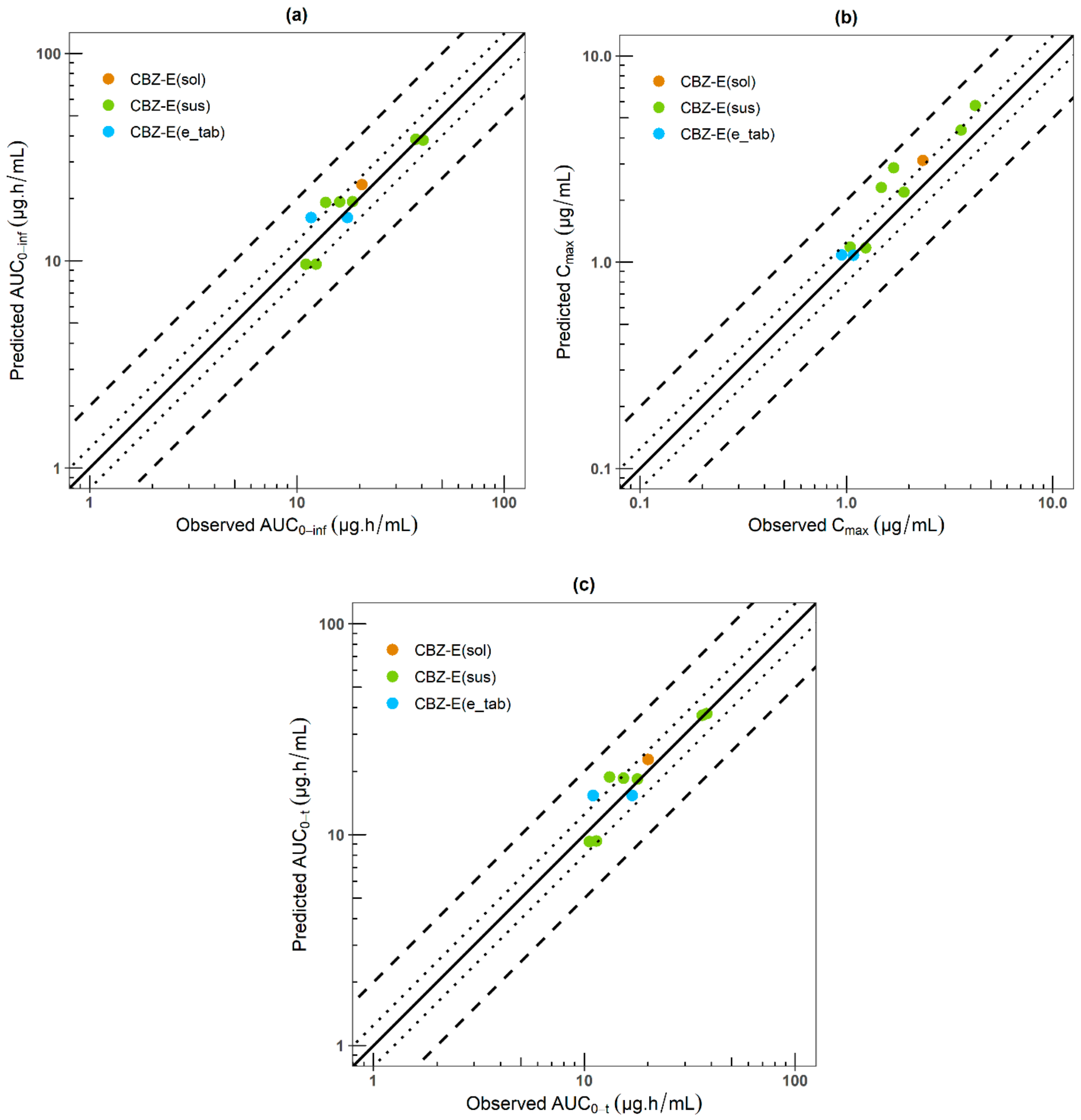

Figure S3 goodness of fit plots for CBZ-E PBPK model performance.
Predicted compared to observed CBZ-E (a)$AUC_{0\text{-}inf}$, (b) C_{max} and (c)$AUC_{0\text{-}t}$ values after oral administration of CBZ-E. The line of identity is shown as a solid line; 1.25-fold deviation is shown as a dotted line; 2-fold deviation is shown as a dashed line.

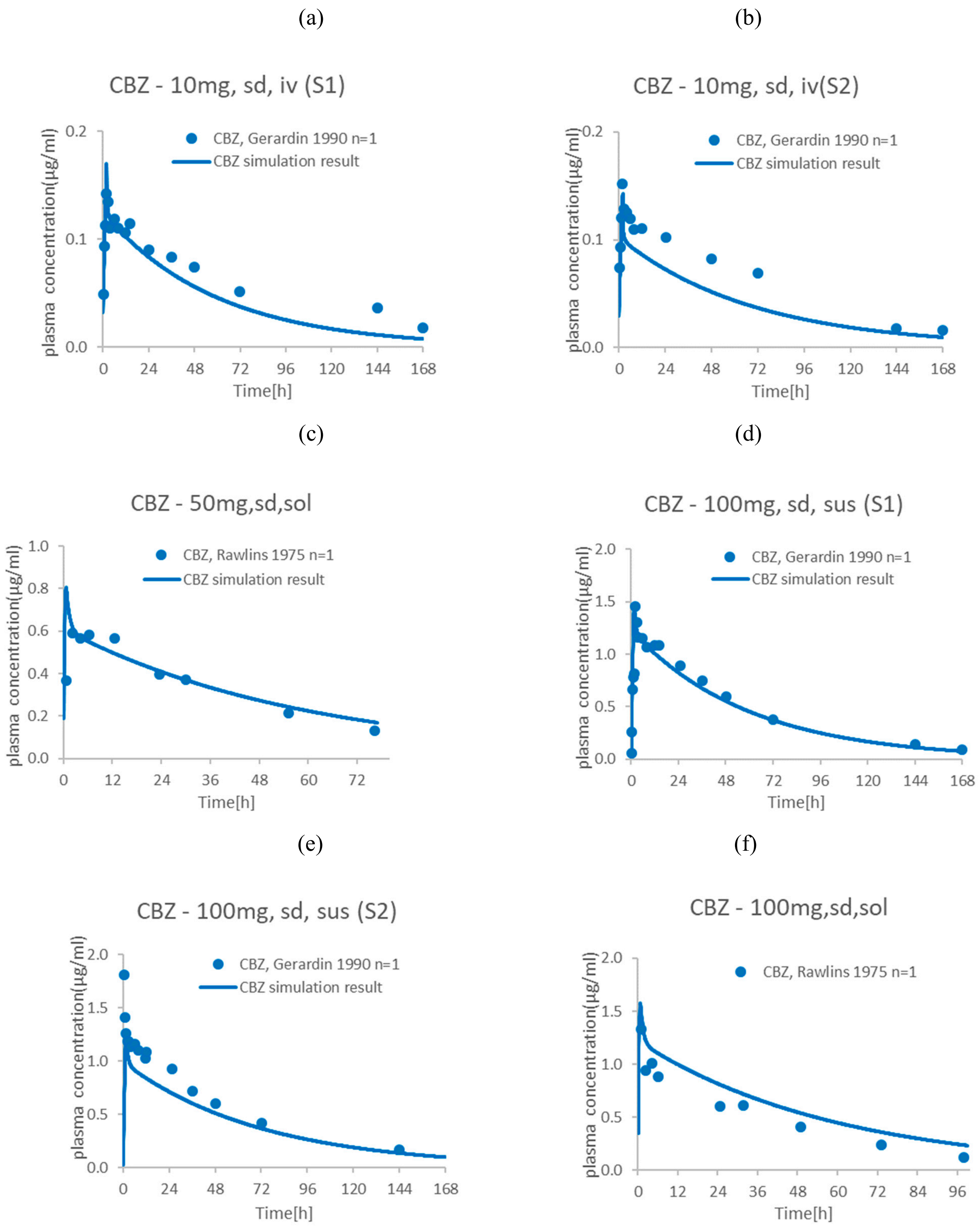

Figure S4 Predicted compared to observed CBZ and CBZ-E plasma concentration-time profiles (linear) after intravenous and oral administration of CBZ.

Observed data are shown as dots ± standard deviation (if applicable); model predictions are shown as solid lines. sd: single dose; iv: intravenous; sol: solution; sus: suspension; tab: immediate-release tablet. CR-tab: control-release tablet; CR-cap: control-release capsule.

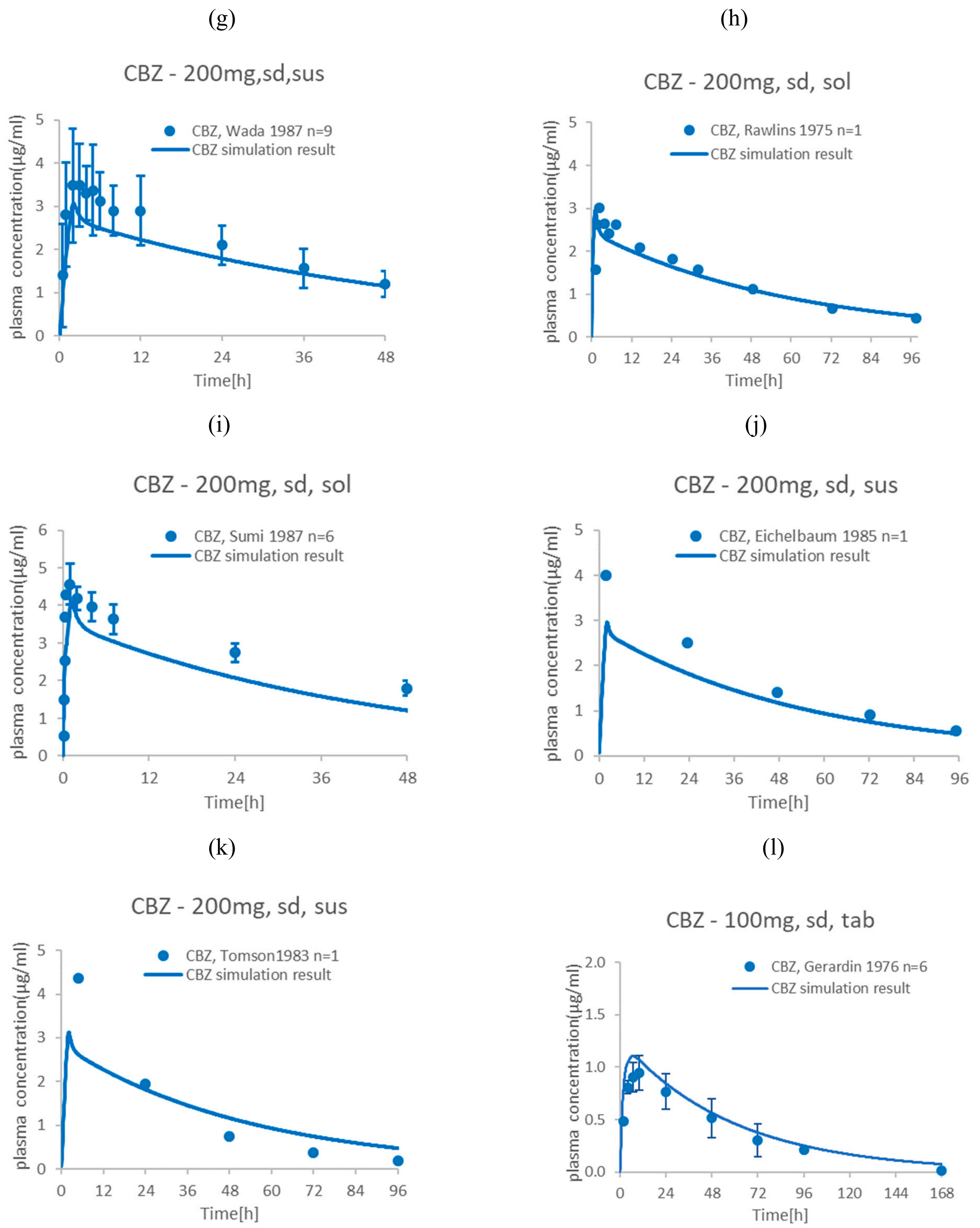

Figure S4 Predicted compared to observed CBZ and CBZ-E plasma concentration-time profiles (linear) after intravenous and oral administration of CBZ*(continued)*.
Observed data are shown as dots ± standard deviation (if applicable); model predictions are shown as solid lines.
sd: single dose; iv: intravenous; sol: solution; sus: suspension; tab: immediate-release tablet. CR-tab: control-release tablet; CR-cap: control-release capsule.

(m)

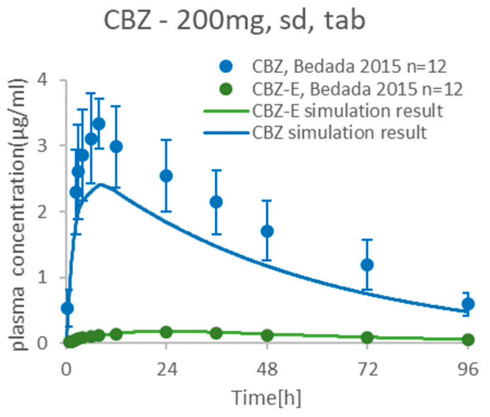

(n)

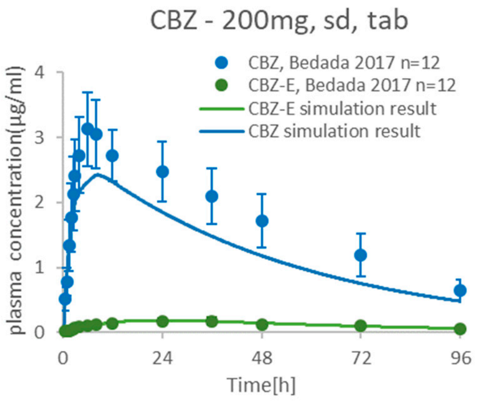

(o)

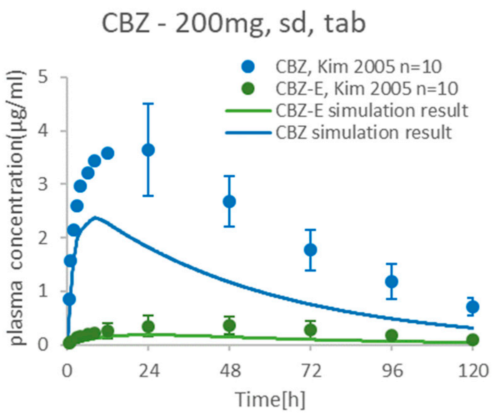

(p)

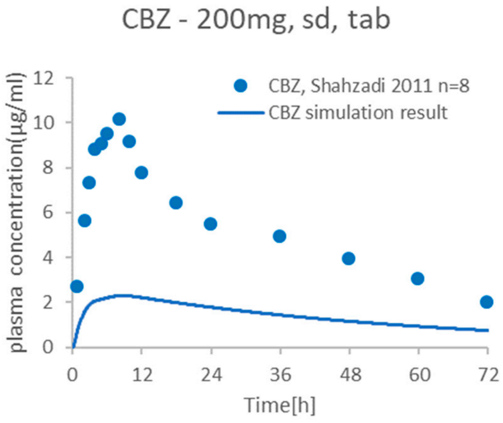

(q)

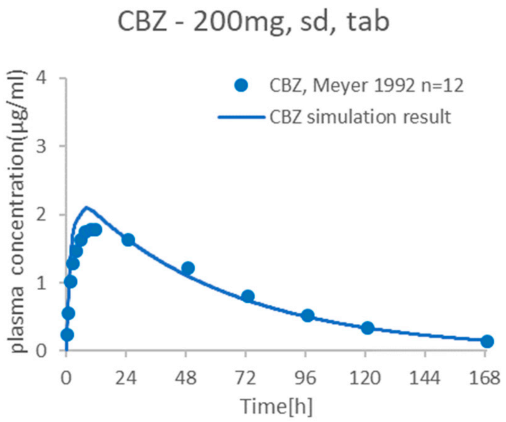

(r)

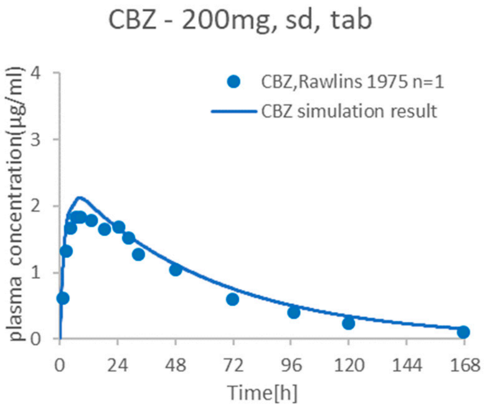

Figure S4 Predicted compared to observed CBZ and CBZ-E plasma concentration-time profiles (linear) after intravenous and oral administration of CBZ*(continued)*.
Observed data are shown as dots ± standard deviation (if applicable); model predictions are shown as solid lines. sd: single dose; iv: intravenous; sol: solution; sus: suspension; tab: immediate-release tablet. CR-tab: control-release tablet; CR-cap: control-release capsule.

(s)

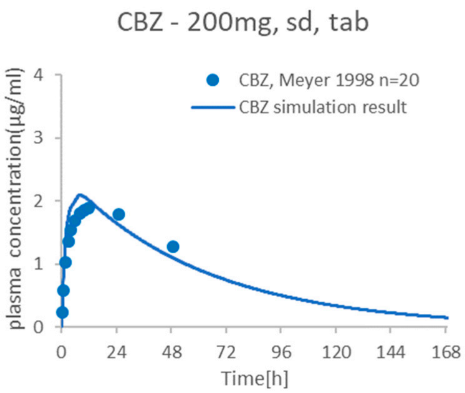

(t)

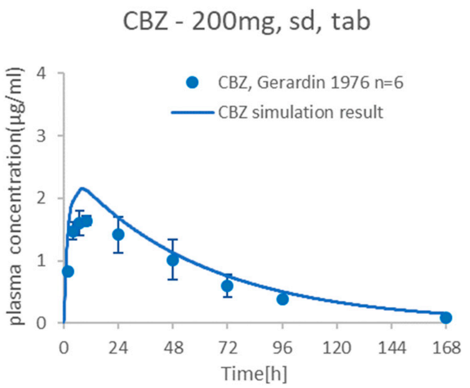

(u)

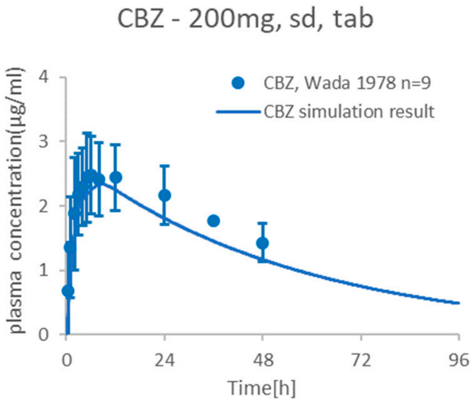

(v)

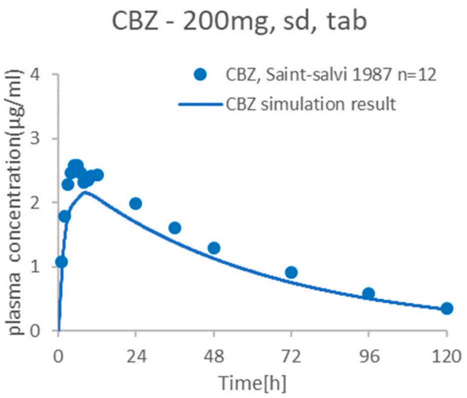

(w)

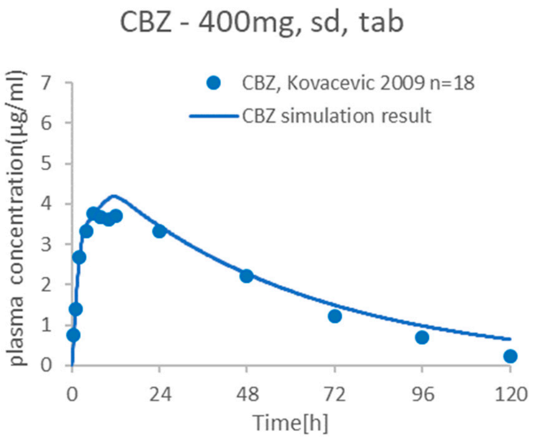

(x)

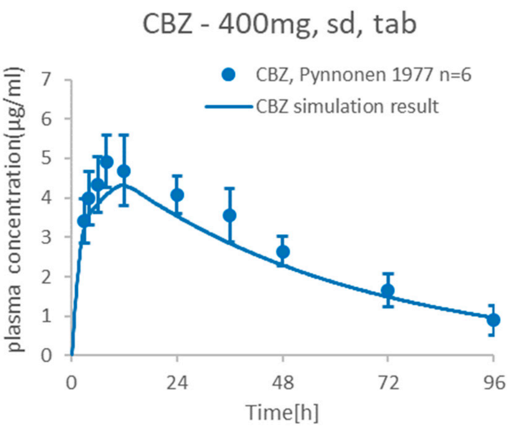

Figure S4 Predicted compared to observed CBZ and CBZ-E plasma concentration-time profiles (linear) after intravenous and oral administration of CBZ*(continued)*.
Observed data are shown as dots ± standard deviation (if applicable); model predictions are shown as solid lines. sd: single dose; iv: intravenous; sol: solution; sus: suspension; tab: immediate-release tablet. CR-tab: control-release tablet; CR-cap: control-release capsule.

(y)

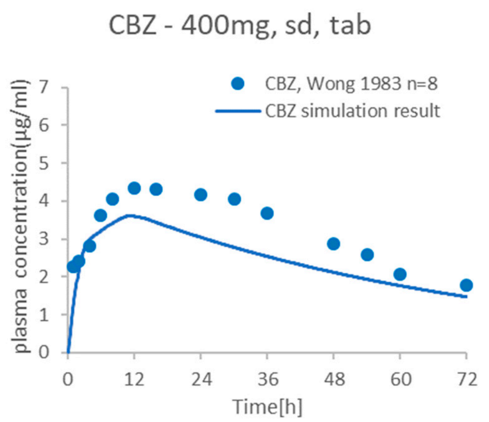

(z)

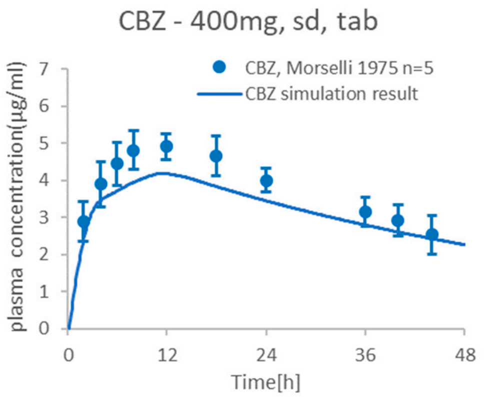

(aa)

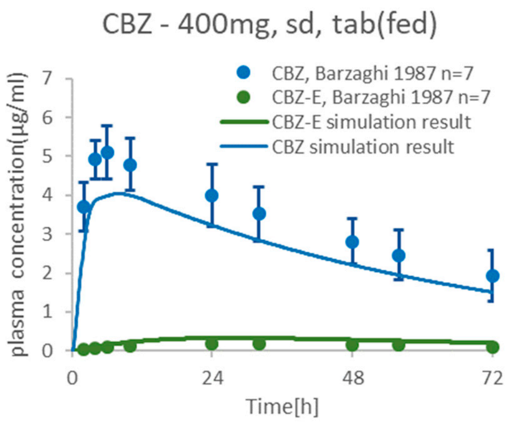

(ab)

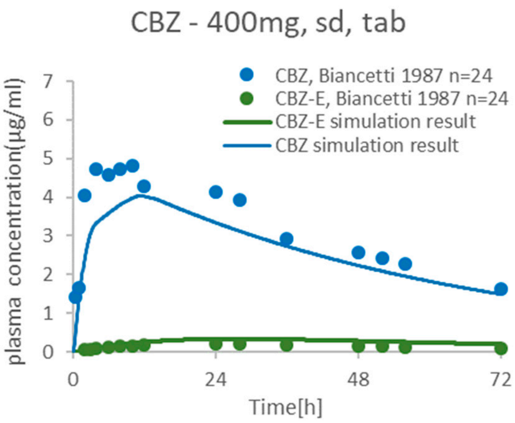

(ac)

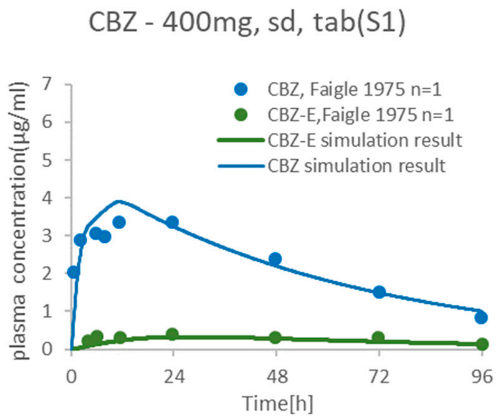

(ad)

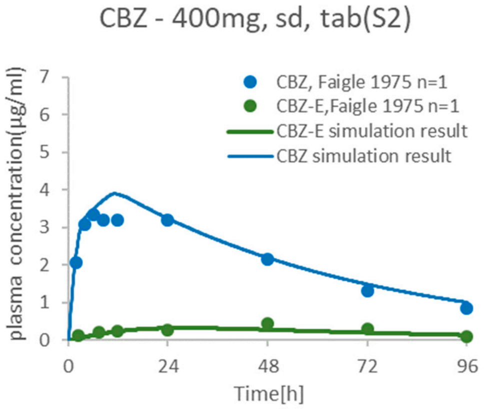

Figure S4 Predicted compared to observed CBZ and CBZ-E plasma concentration-time profiles (linear) after intravenous and oral administration of CBZ*(continued)*.
Observed data are shown as dots ± standard deviation (if applicable); model predictions are shown as solid lines.
sd: single dose; iv: intravenous; sol: solution; sus: suspension; tab: immediate-release tablet. CR-tab: control-release tablet; CR-cap: control-release capsule.

(ae)

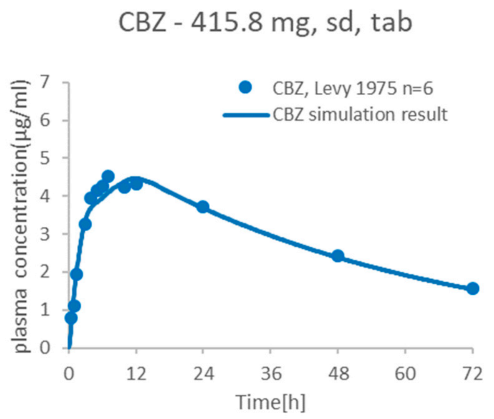

(af)

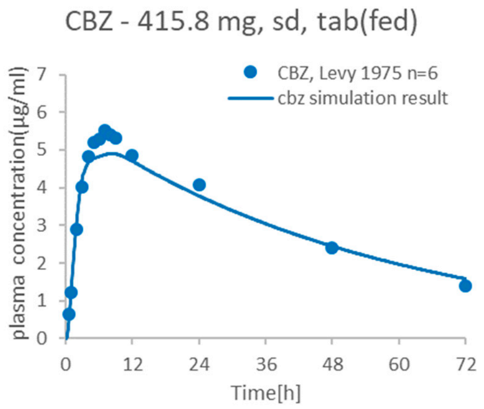

(ag)

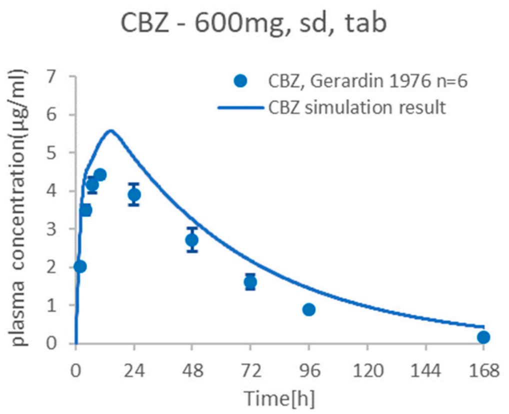

(ah)

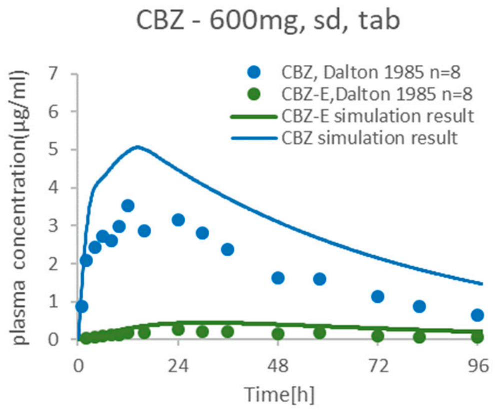

(ai)

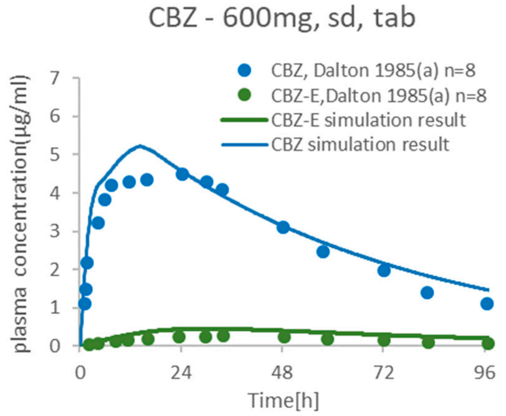

(aj)

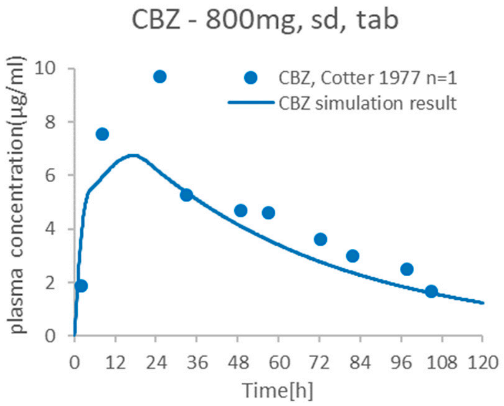

Figure S4 Predicted compared to observed CBZ and CBZ-E plasma concentration-time profiles (linear) after intravenous and oral administration of CBZ*(continued)*.
Observed data are shown as dots ± standard deviation (if applicable); model predictions are shown as solid lines. sd: single dose; iv: intravenous; sol: solution; sus: suspension; tab: immediate-release tablet. CR-tab: control-release tablet; CR-cap: control-release capsule.

(ak)

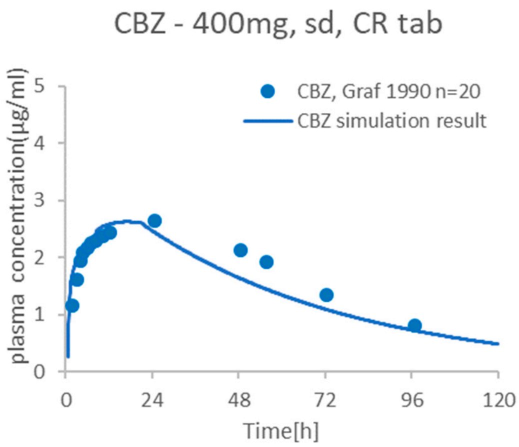

(al)

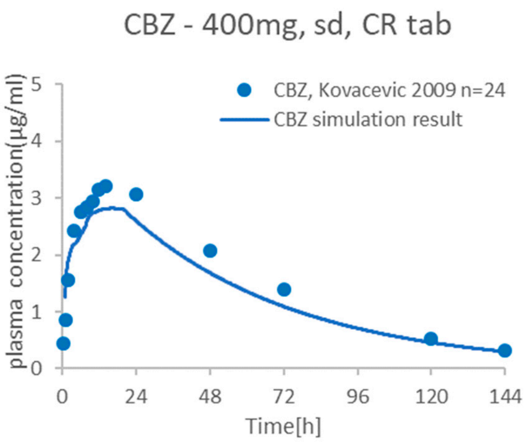

(am)

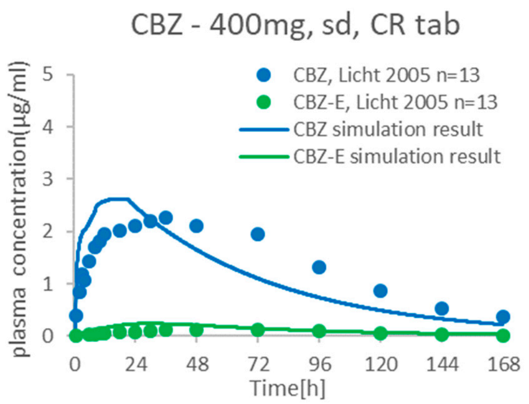

(an)

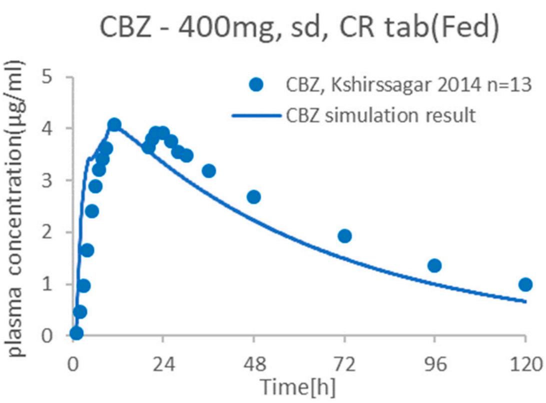

(ao)

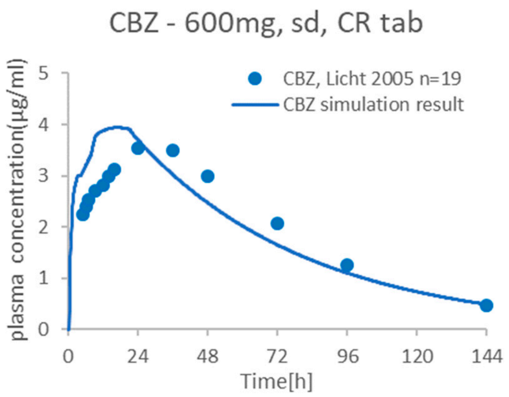

(ap)

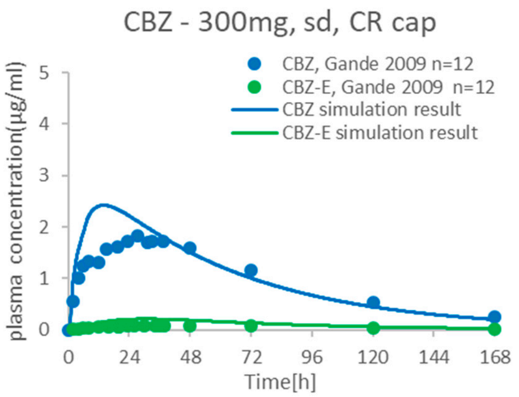

Figure S4 Predicted compared to observed CBZ and CBZ-E plasma concentration-time profiles (linear) after intravenous and oral administration of CBZ*(continued)*.
Observed data are shown as dots ± standard deviation (if applicable); model predictions are shown as solid lines. sd: single dose; iv: intravenous; sol: solution; sus: suspension; tab: immediate-release tablet. CR-tab: control-release tablet; CR-cap: control-release capsule.

(aq)

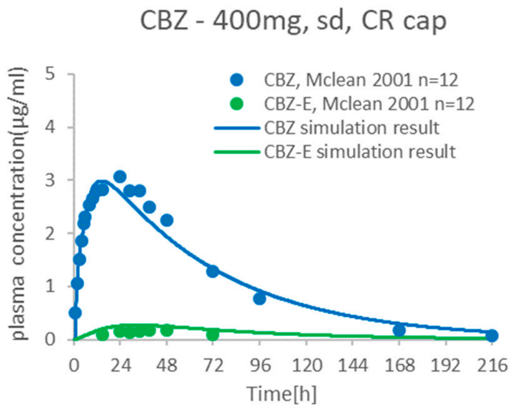

(ar)

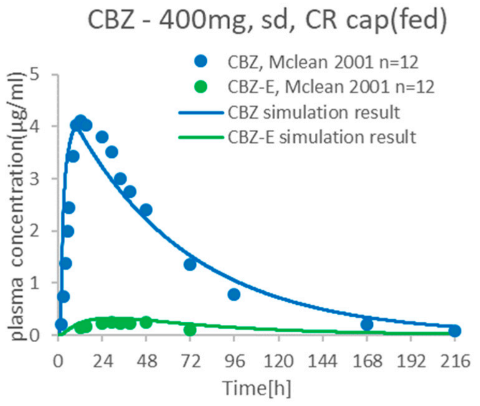

Figure S4 Predicted compared to observed CBZ and CBZ-E plasma concentration-time profiles (linear) after intravenous and oral administration of CBZ*(continued)*.

Observed data are shown as dots ± standard deviation (if applicable); model predictions are shown as solid lines. sd: single dose; iv: intravenous; sol: solution; sus: suspension; tab: immediate-release tablet. CR-tab: control-release tablet; CR-cap: control-release capsule.

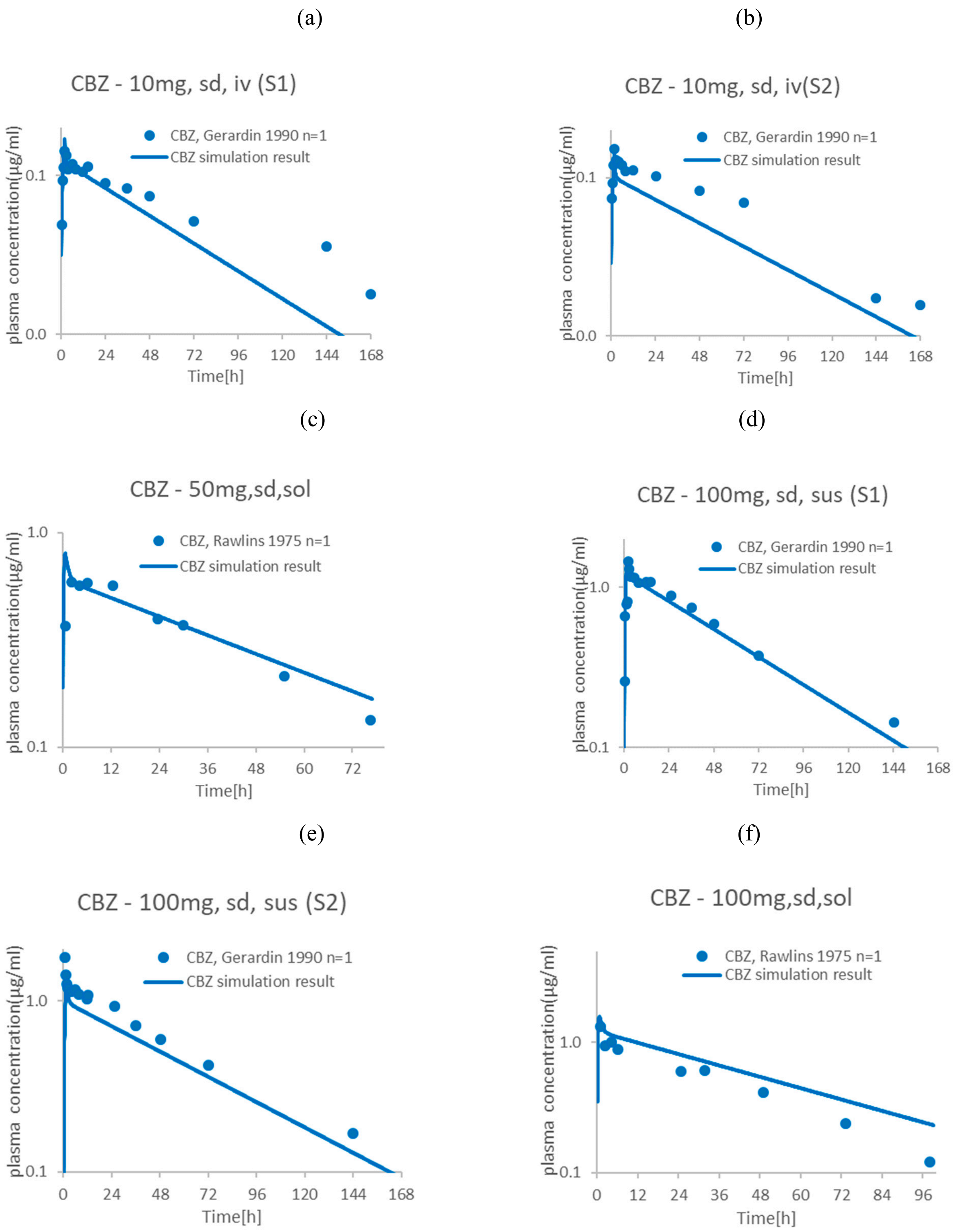

Figure S5 Predicted compared to observed CBZ and CBZ-E plasma concentration-time profiles (Semi-logarithmic) after intravenous and oral administration of CBZ.
Observed data are shown as dots ± standard deviation (if applicable); model predictions are shown as solid lines. sd: single dose; iv: intravenous; sol: solution; sus: suspension; tab: immediate-release tablet. CR-tab: control-release tablet; CR-cap: control-release capsule.

(g)

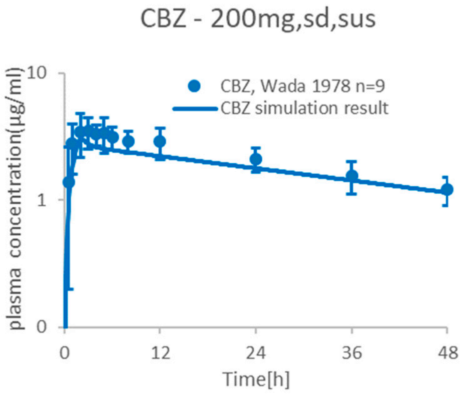

(h)

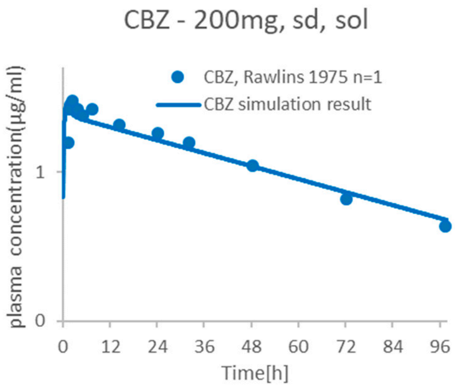

(i)

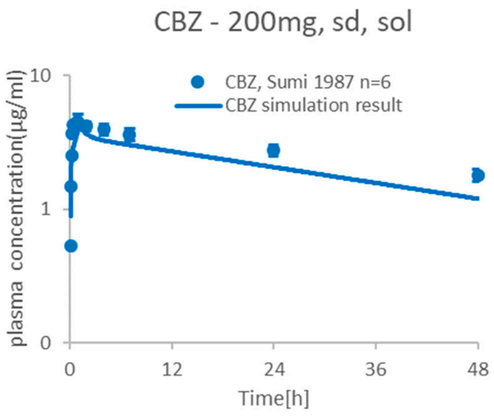

(j)

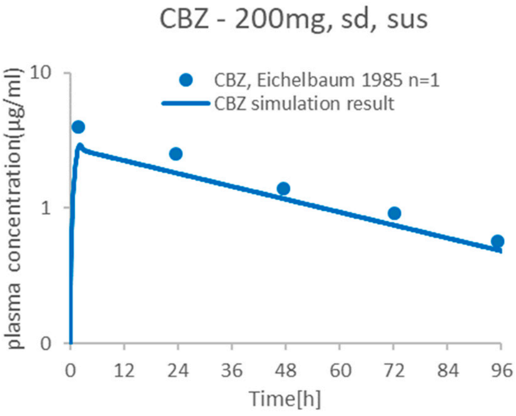

(k)

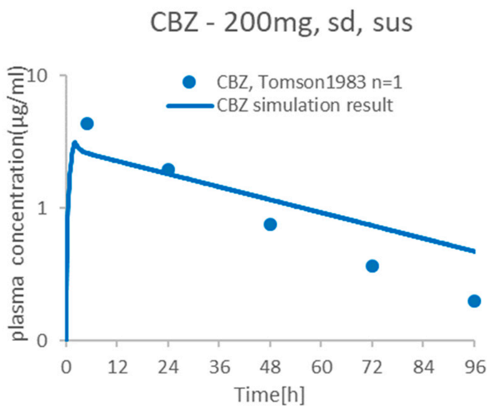

(l)

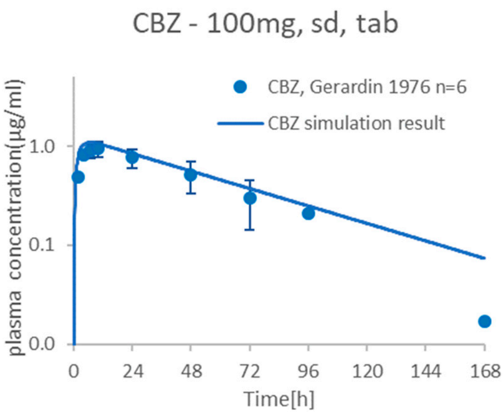

Figure S5 Predicted compared to observed CBZ and CBZ-E plasma concentration-time profiles (Semi-logarithmic) after intravenous and oral administration of CBZ *(continued)*.
Observed data are shown as dots ± standard deviation (if applicable); model predictions are shown as solid lines. sd: single dose; iv: intravenous; sol: solution; sus: suspension; tab: immediate-release tablet. CR-tab: control-release tablet; CR-cap: control-release capsule.

(m)

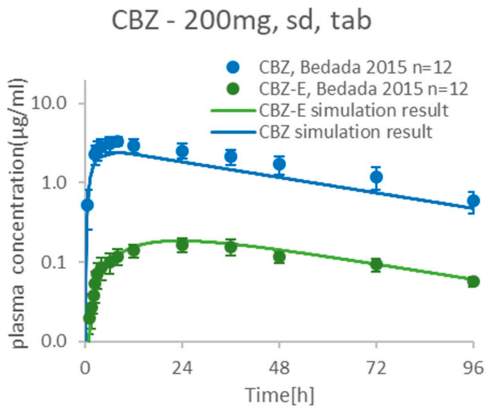

(n)

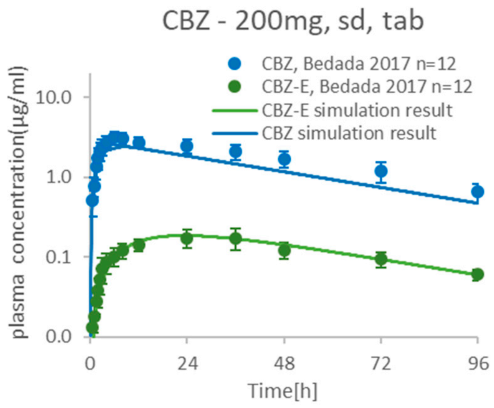

(o)

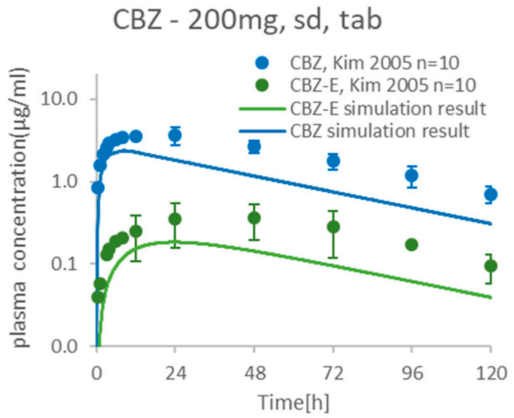

(p)

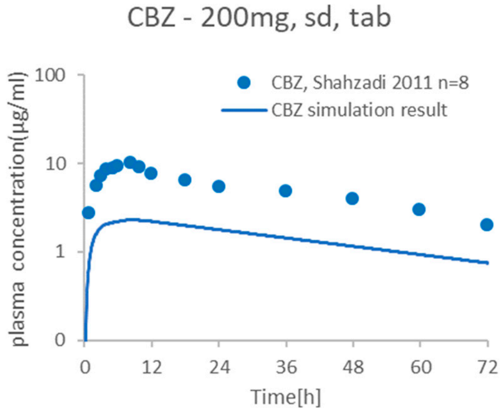

(q)

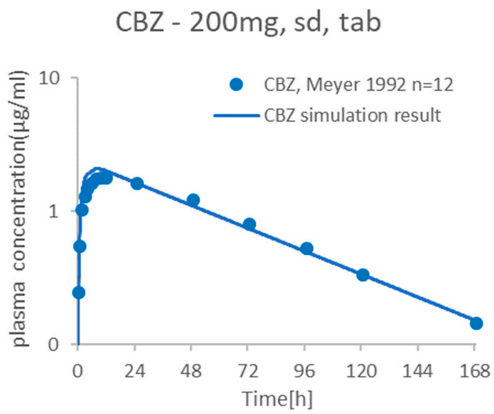

(r)

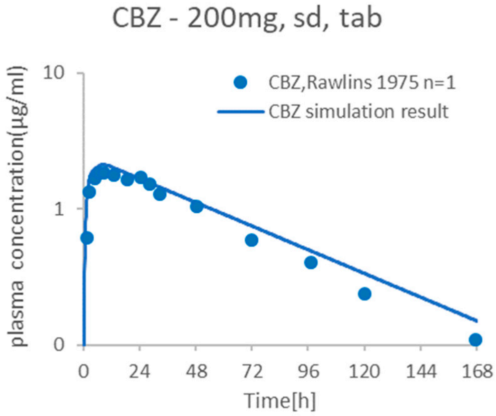

Figure S5 Predicted compared to observed CBZ and CBZ-E plasma concentration-time profiles (Semi-logarithmic) after intravenous and oral administration of CBZ *(continued)*.
Observed data are shown as dots ± standard deviation (if applicable); model predictions are shown as solid lines. sd: single dose; iv: intravenous; sol: solution; sus: suspension; tab: immediate-release tablet. CR-tab: control-release tablet; CR-cap: control-release capsule.

(s)

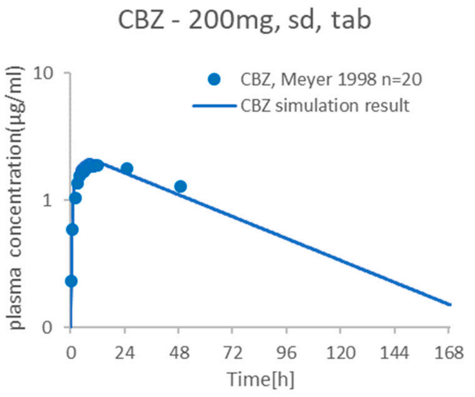

(t)

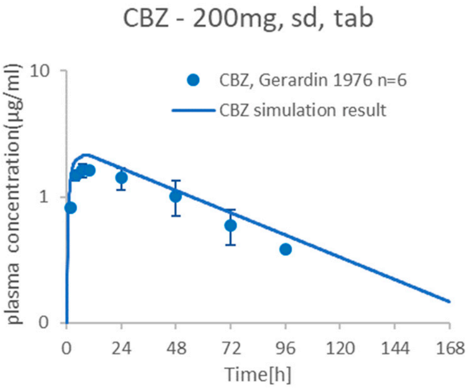

(u)

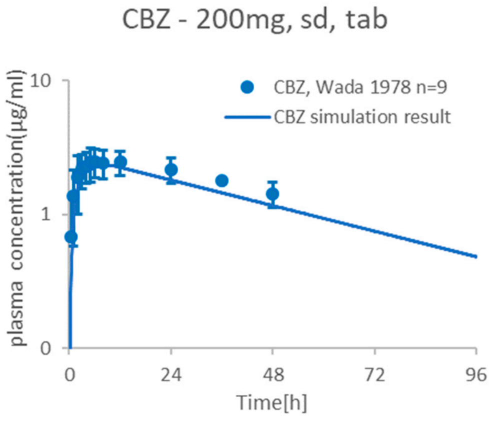

(v)

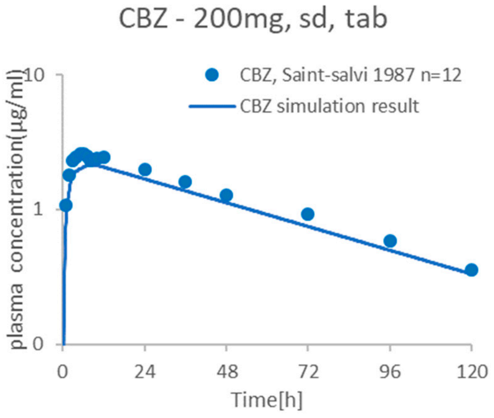

(w)

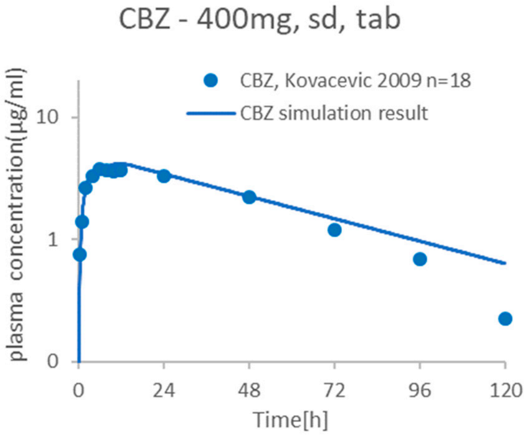

(x)

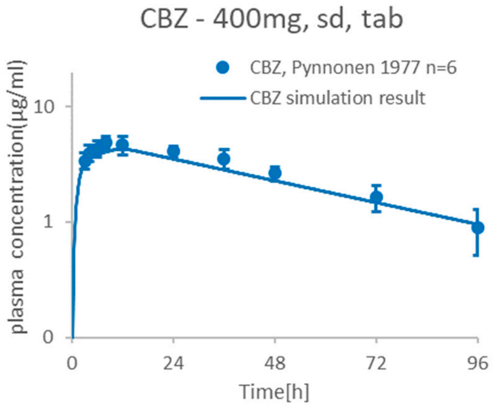

Figure S5 Predicted compared to observed CBZ and CBZ-E plasma concentration-time profiles (Semi-logarithmic) after intravenous and oral administration of CBZ *(continued)*.
Observed data are shown as dots ± standard deviation (if applicable); model predictions are shown as solid lines. sd: single dose; iv: intravenous; sol: solution; sus: suspension; tab: immediate-release tablet. CR-tab: control-release tablet; CR-cap: control-release capsule.

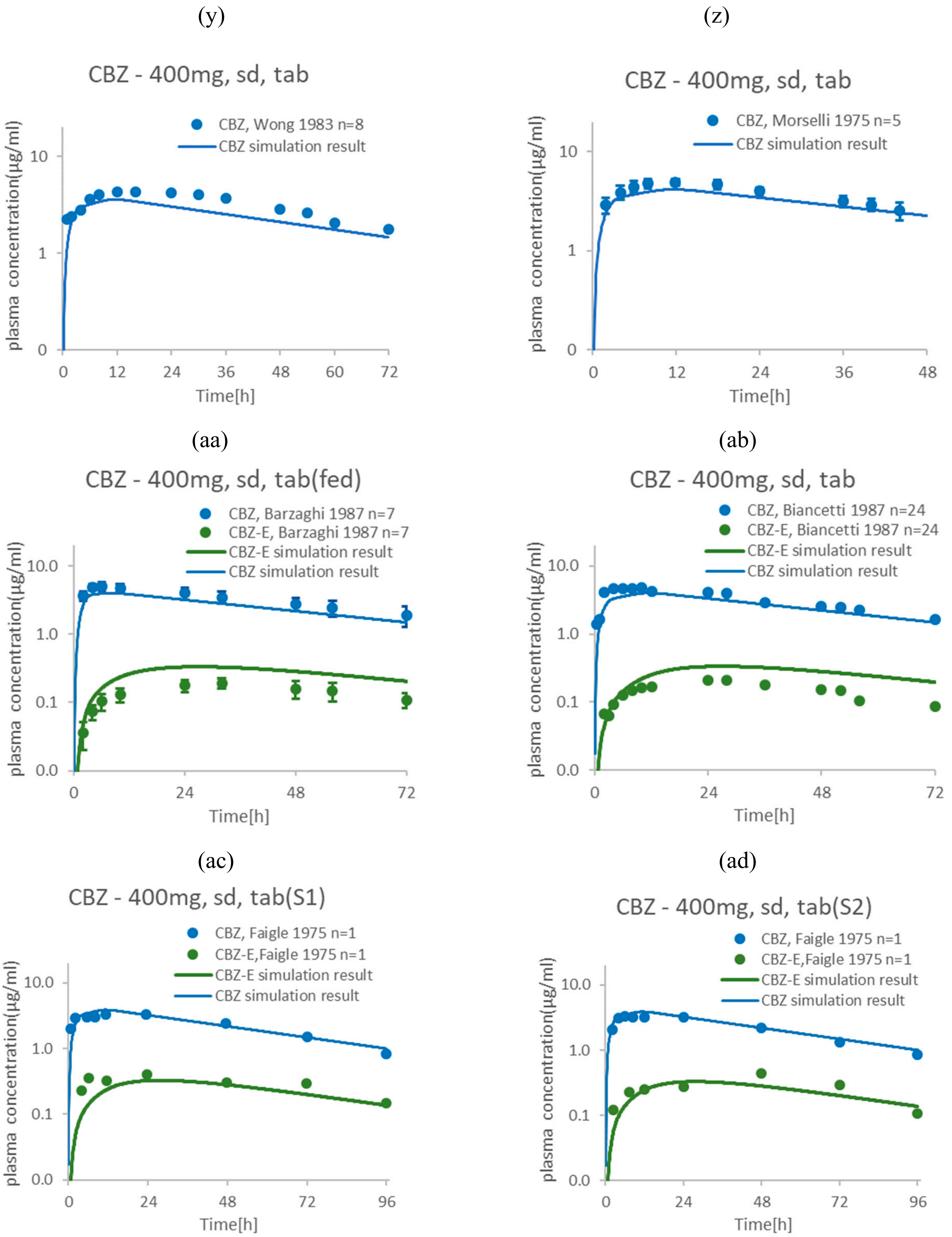

Figure S5 Predicted compared to observed CBZ and CBZ-E plasma concentration-time profiles (Semi-logarithmic) after intravenous and oral administration of CBZ *(continued)*.
Observed data are shown as dots ± standard deviation (if applicable); model predictions are shown as solid lines. sd: single dose; iv: intravenous; sol: solution; sus: suspension; tab: immediate-release tablet. CR-tab: control-release tablet; CR-cap: control-release capsule.

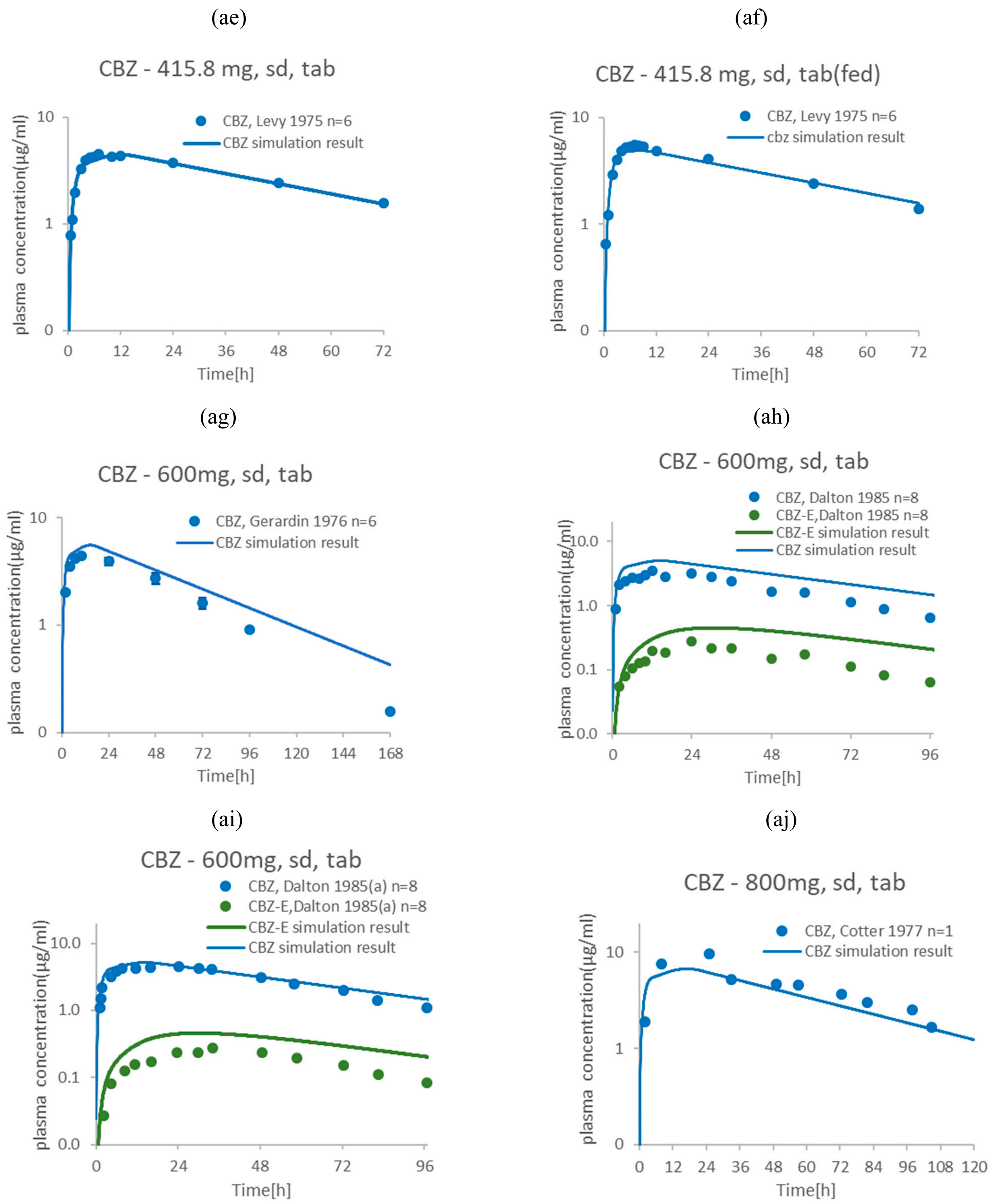

Figure S5 Predicted compared to observed CBZ and CBZ-E plasma concentration-time profiles (Semi-logarithmic) after intravenous and oral administration of CBZ *(continued)*.
Observed data are shown as dots ± standard deviation (if applicable); model predictions are shown as solid lines. sd: single dose; iv: intravenous; sol: solution; sus: suspension; tab: immediate-release tablet. CR-tab: control-release tablet; CR-cap: control-release capsule.

(ak)

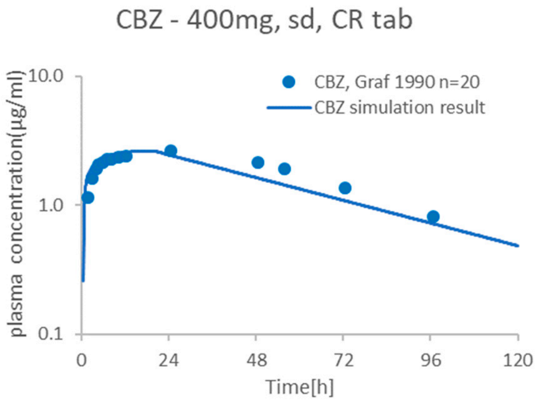

(al)

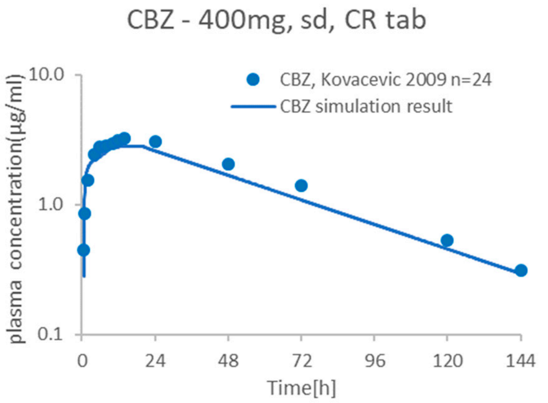

(am)

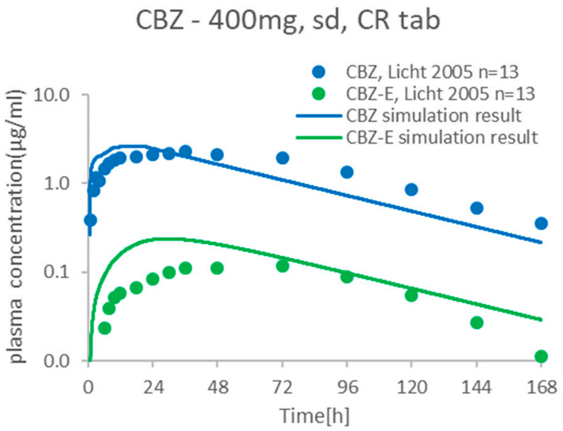

(an)

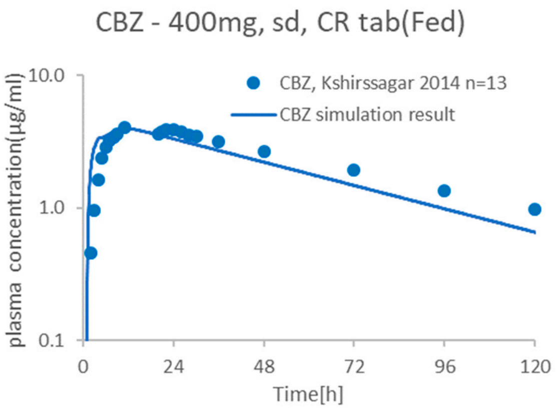

(ao)

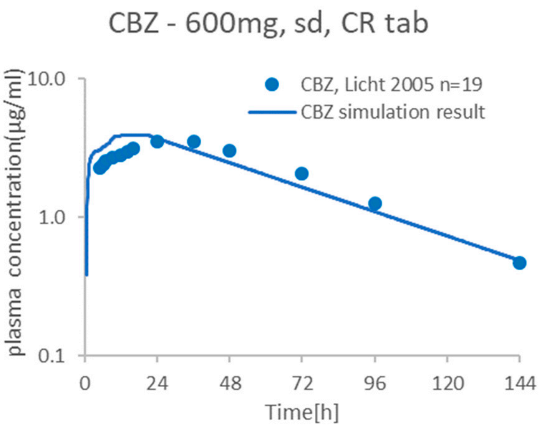

(ap)

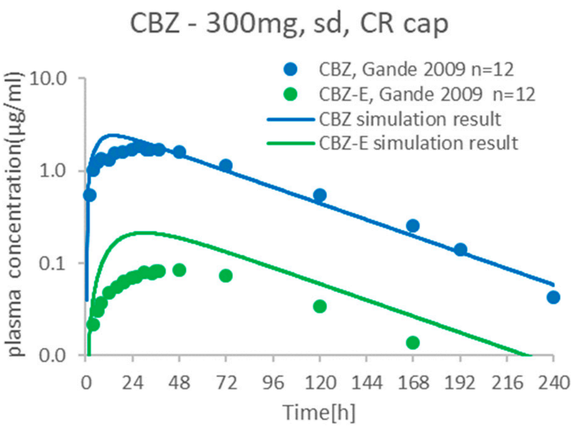

Figure S5 Predicted compared to observed CBZ and CBZ-E plasma concentration-time profiles (Semi-logarithmic) after intravenous and oral administration of CBZ *(continued)*.
Observed data are shown as dots ± standard deviation (if applicable); model predictions are shown as solid lines. sd: single dose; iv: intravenous; sol: solution; sus: suspension; tab: immediate-release tablet. CR-tab: control-release tablet; CR-cap: control-release capsule.

(aq) (ar)

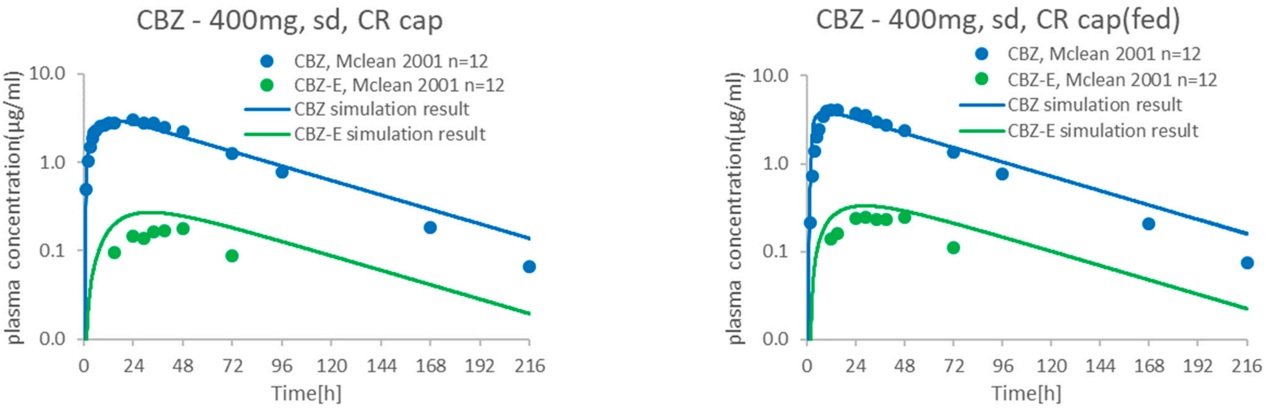

Figure S5 Predicted compared to observed CBZ and CBZ-E plasma concentration-time profiles (Semi-logarithmic) after intravenous and oral administration of CBZ *(continued)*.
Observed data are shown as dots ± standard deviation (if applicable); model predictions are shown as solid lines.
sd: single dose; iv: intravenous; sol: solution; sus: suspension; tab: immediate-release tablet. CR-tab: control-release tablet; CR-cap: control-release capsule.

(a) (b)

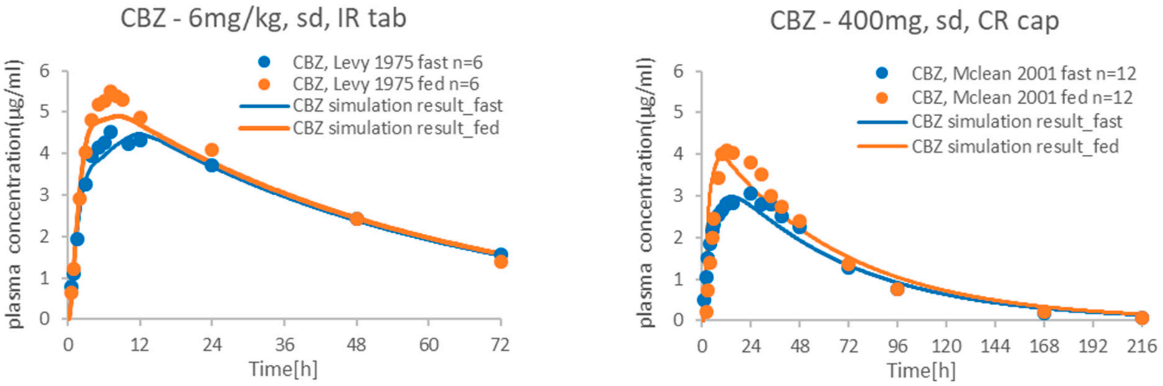

Figure S6 Model predictions comparison of CBZ concentration-time profiles in fast and fed conditions.
(a) After 6mg/kg single-dose immediate-release tablet; (b)After 400mg single-dose control-release capsule.
Observed data are shown as dots ± standard deviation (if applicable); model predictions are shown as solid lines.
sd: single dose; tab: immediate-release tablet. CR-cap: control-release capsule.

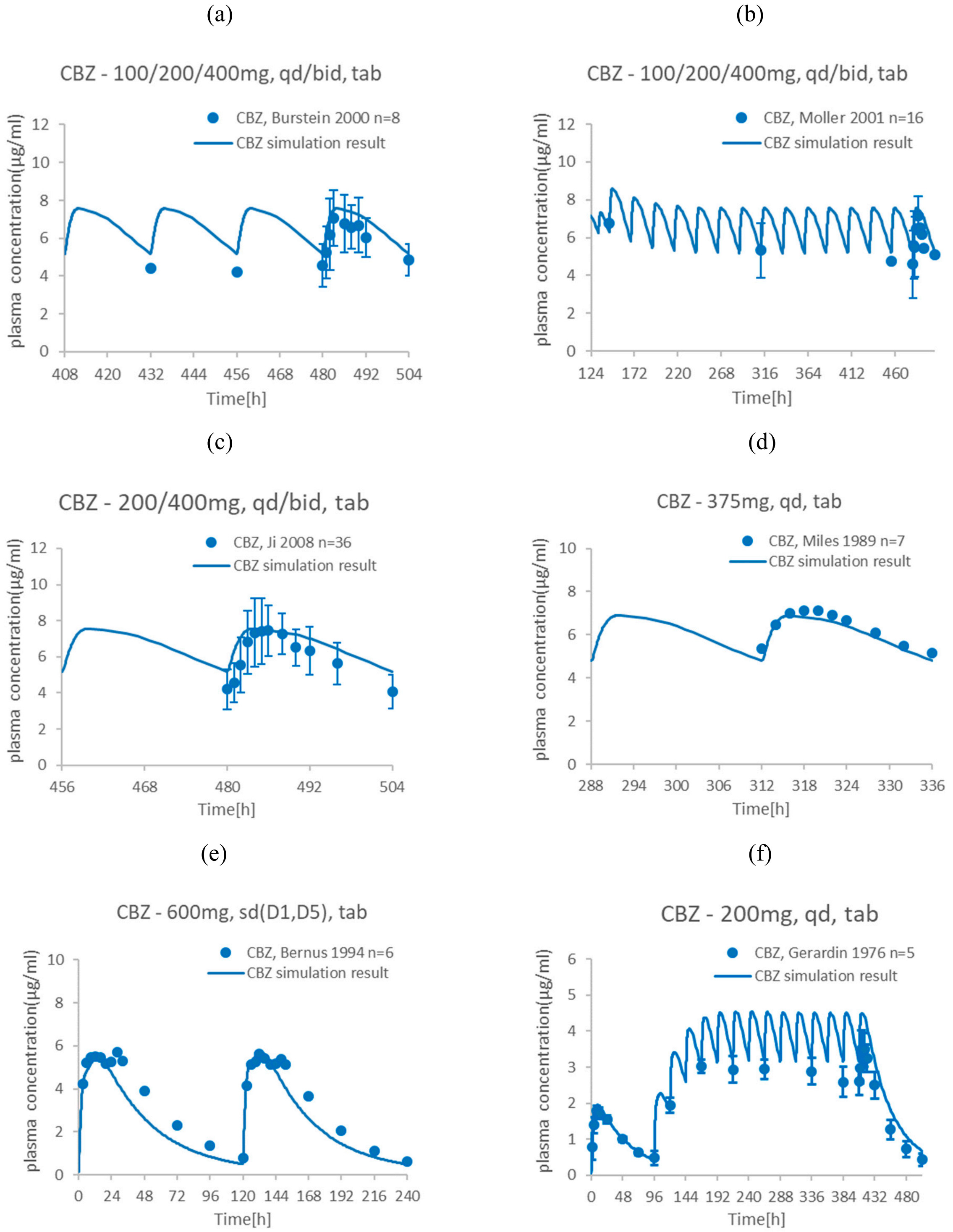

Figure S7 Predicted compared to observed CBZ plasma concentration-time profiles(linear) after oral administration of CBZ.

Observed data are shown as dots ± standard deviation (if applicable); model predictions are shown as solid lines. sd: single dose; tab: immediate-release tablet; qd: once daily; bid: twice times daily; tid: three times daily.

(g)

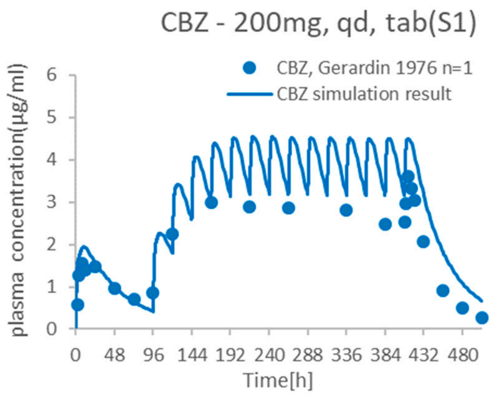

(h)

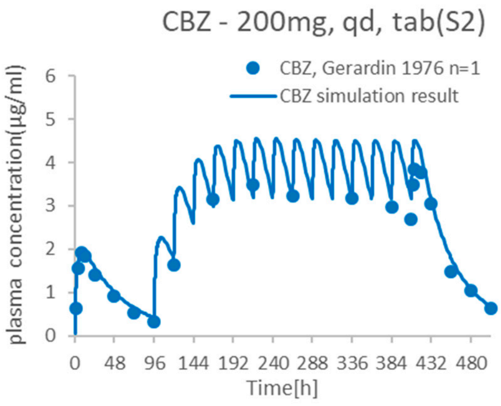

(i)

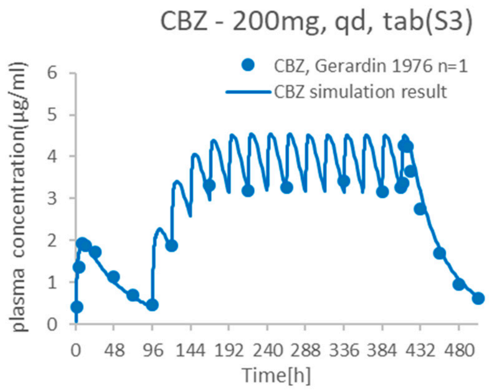

(j)

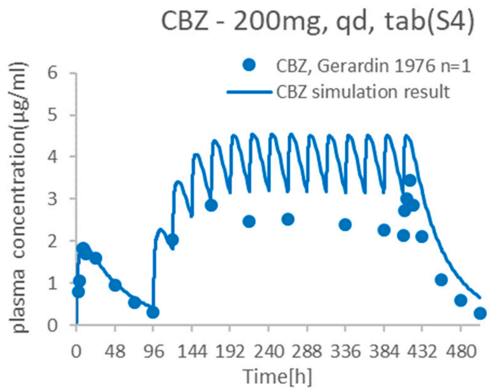

(k)

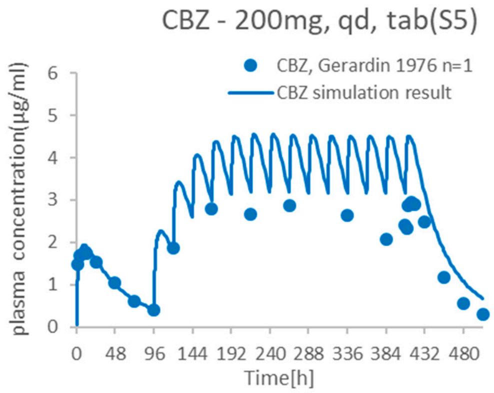

Figure S7 Predicted compared to observed CBZ plasma concentration-time profiles(linear) after oral administration of CBZ *(continued)*.

Observed data are shown as dots ± standard deviation (if applicable); model predictions are shown as solid lines. sd: single dose; tab: immediate-release tablet; qd: once daily; bid: twice times daily; tid: three times daily.

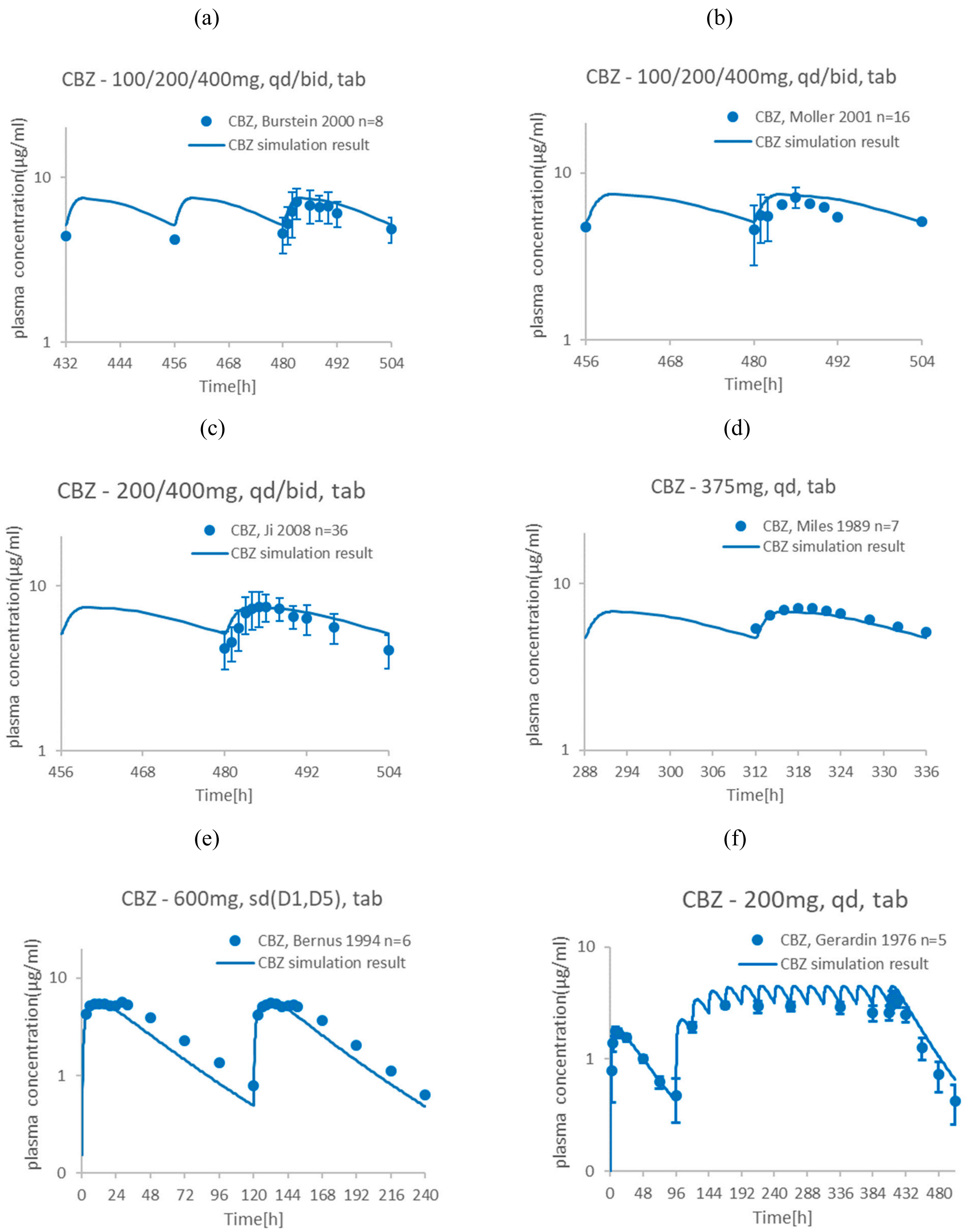

Figure S8 Predicted compared to observed CBZ plasma concentration-time profiles (Semi-logarithmic) after oral administration of CBZ.

Observed data are shown as dots ± standard deviation (if applicable); model predictions are shown as solid lines. sd: single dose; tab: immediate-release tablet; qd: once daily; bid: twice times daily; tid: three times daily.

(g)

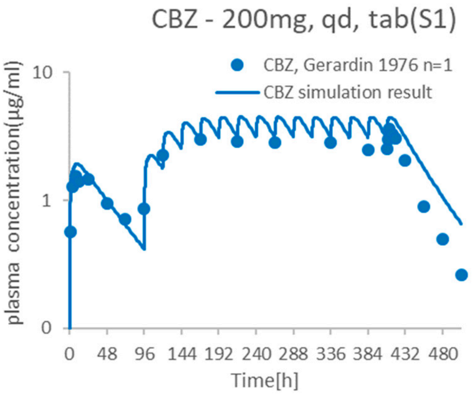

(h)

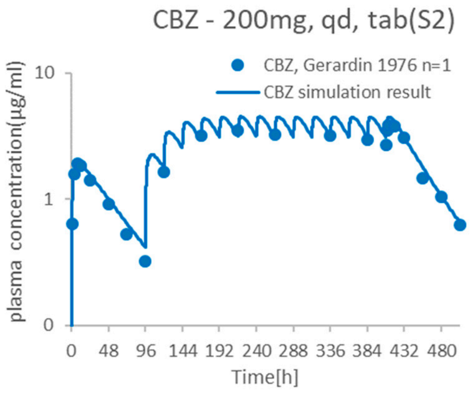

(i)

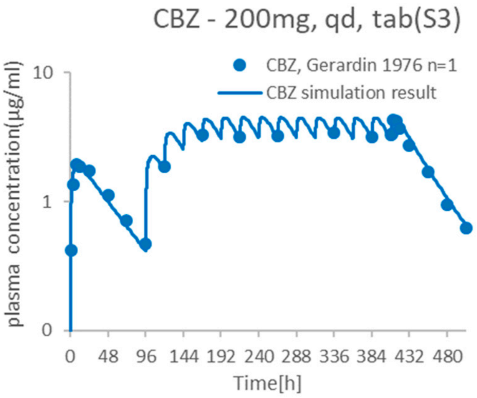

(j)

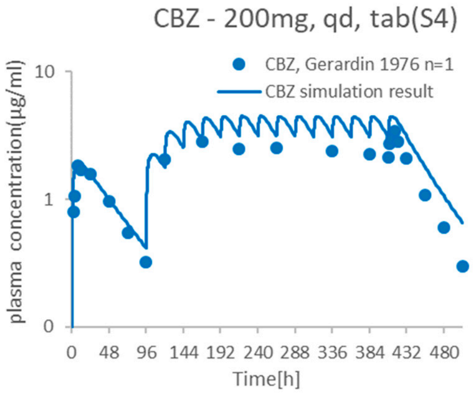

(k)

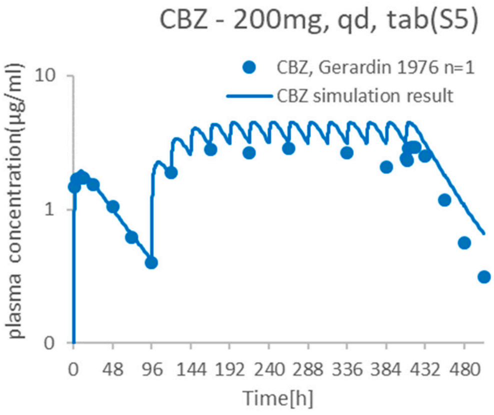

Figure S8 Predicted compared to observed CBZ plasma concentration-time profiles (Semi-logarithmic) after oral administration of CBZ *(continued)*.

Observed data are shown as dots ± standard deviation (if applicable); model predictions are shown as solid lines. sd: single dose; tab: immediate-release tablet; qd: once daily; bid: twice times daily; tid: three times daily.

(a) (b) (c)

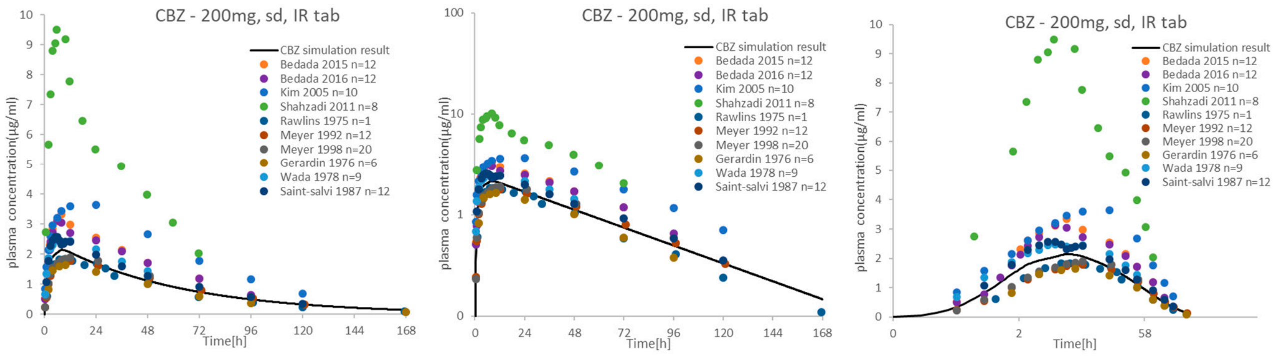

Figure S9 Summary plot for all clinical observed data when given 200mg CBZ IR tablet.
(a) normal scale; (b) y-axis in log scale; (c) x-axis in log scale. The solid line shows the CBZ parent model simulation result for Gerardin 1976 clinical study[9]. Dots represent observed data.

(a) (b) (c)

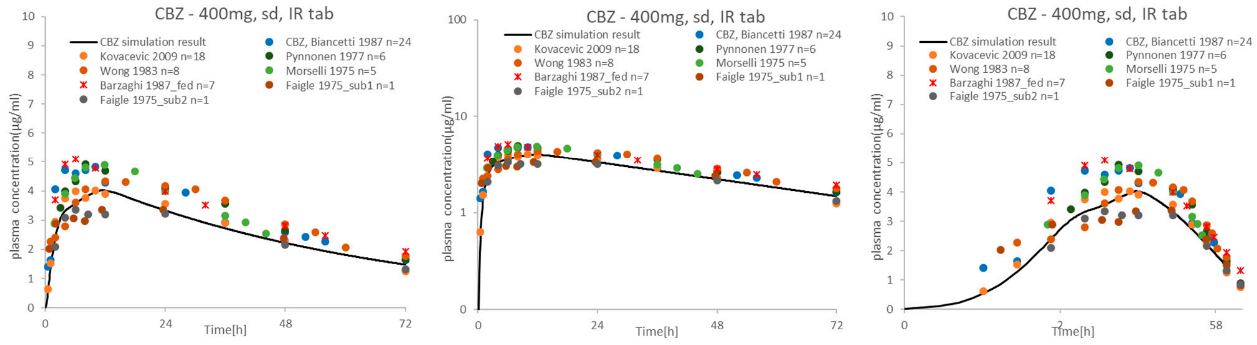

Figure S10 Summary plot for all clinical observed data when given 400mg CBZ IR tablet.
(a) normal scale; (b) y-axis in log scale; (c) x-axis in log scale. The solid line shows the CBZ parent model simulation result for Biancetti 1987 clinical study. Dots represent observed data.

(a) (b) (c)

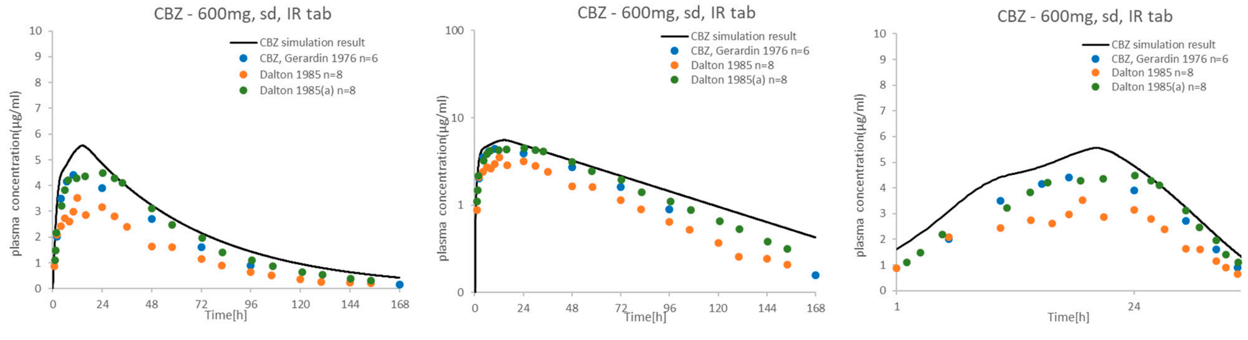

Figure S11 Summary plot for all clinical observed data when given 600mg CBZ IR tablet.
(a) normal scale; (b) y-axis in log scale; (c) x-axis in log scale. The solid line shows the CBZ parent model simulation result for Gerardin 1976 clinical study[9]. Dots represent observed data.

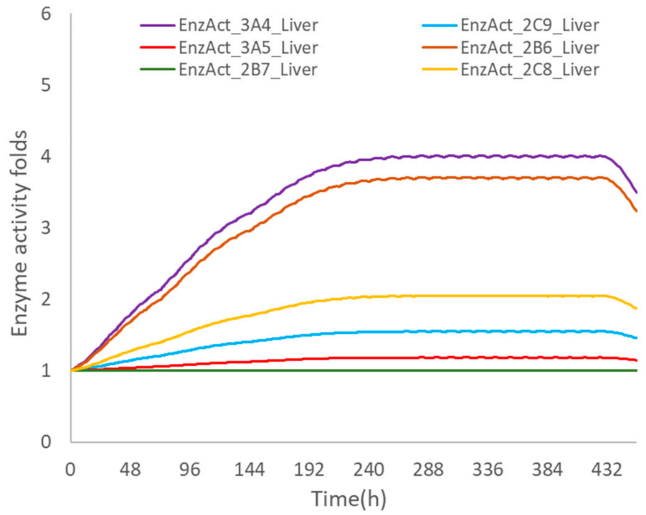

Figure S12 CBZ induction capacity for different enzymes.

CBZ is given 300mg twice times a day for 18 days; EnzAct represents Enzyme activity fold.

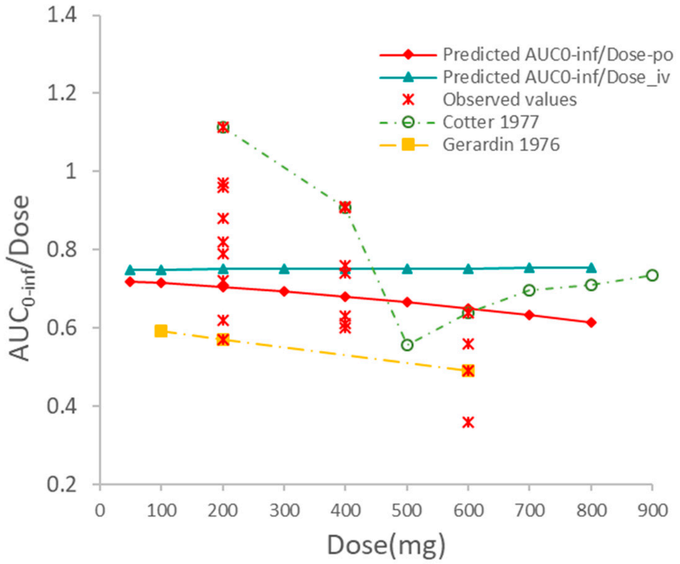

Figure S2 CBZ nonlinear PK exploration for single dose.

Each red solid triangle represents one clinical study. All the AUC0-inf values are calculated based on digitized data except Cotter's 1976 study, in which AUC values were digitized from a plot in the literature.

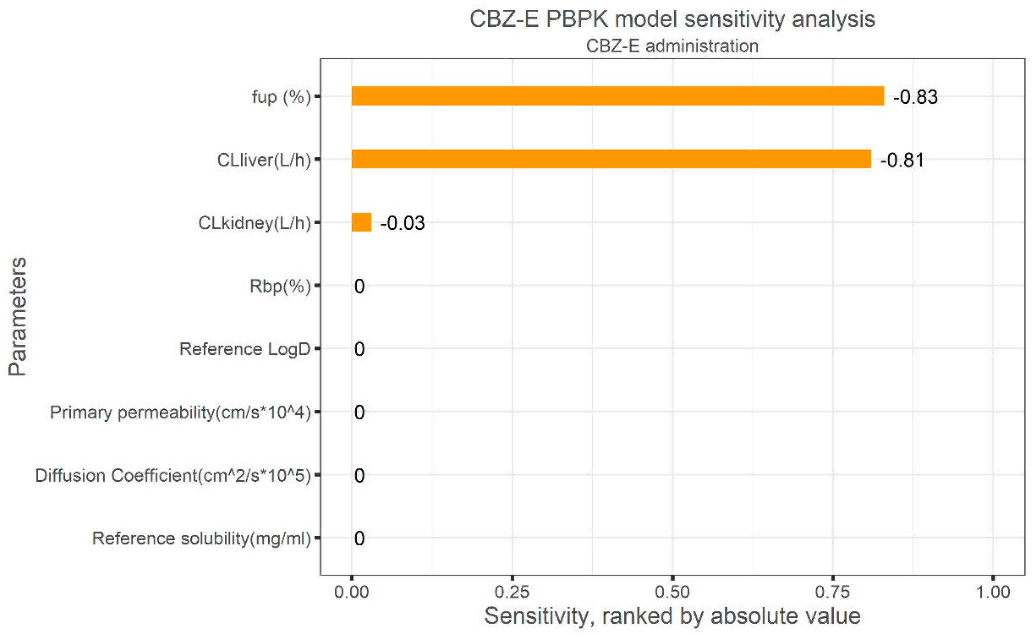

Figure S3 CBZ-E PBPK model sensitivity analysis.

Rbp: blood to plasma ratio; fup: plasma unbound fraction; CL_{liver}: Liver clearance; CL_{kidney}: kidney clearance

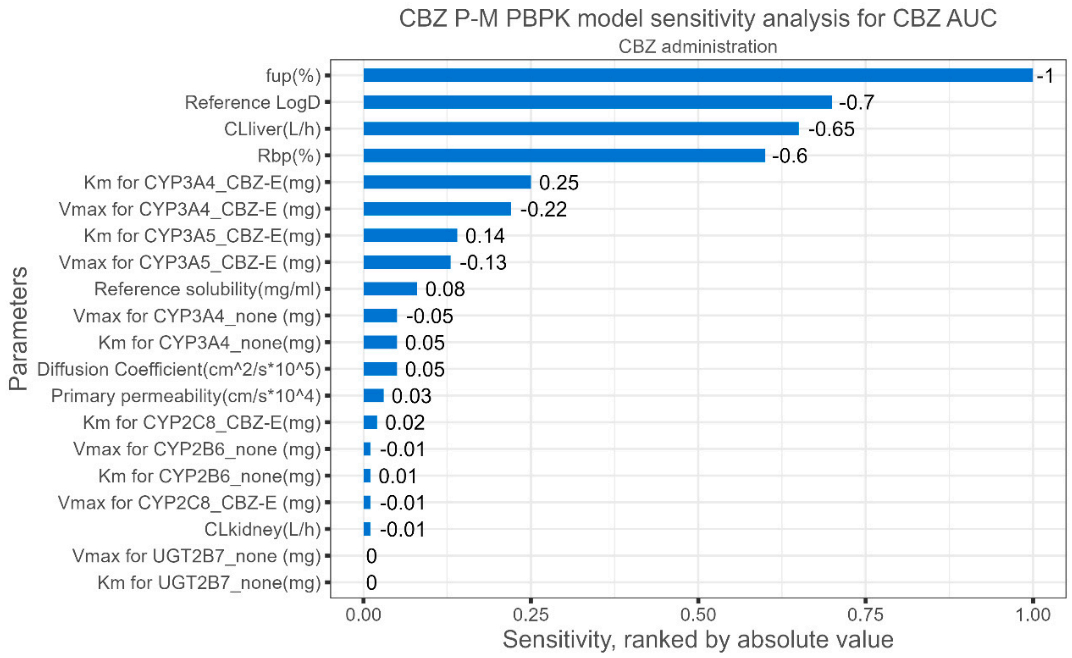

Figure S4 CBZ P-M PBPK model sensitivity analysis calculated as change of the simulated CBZ $AUC_{0\text{-}inf}$.

Vmax: maximal reaction rate; km: Michaelis-Menten constant (half of the maximum reaction rate is achieved); CYP:cytochrome P450; UGT: UDP-glucuronosyltransferanse; Rbp: blood to plasma ratio; fup: plasma unbound fraction; CL_{liver}: Liver clearance; CL_{kidney}: kidney clearance;

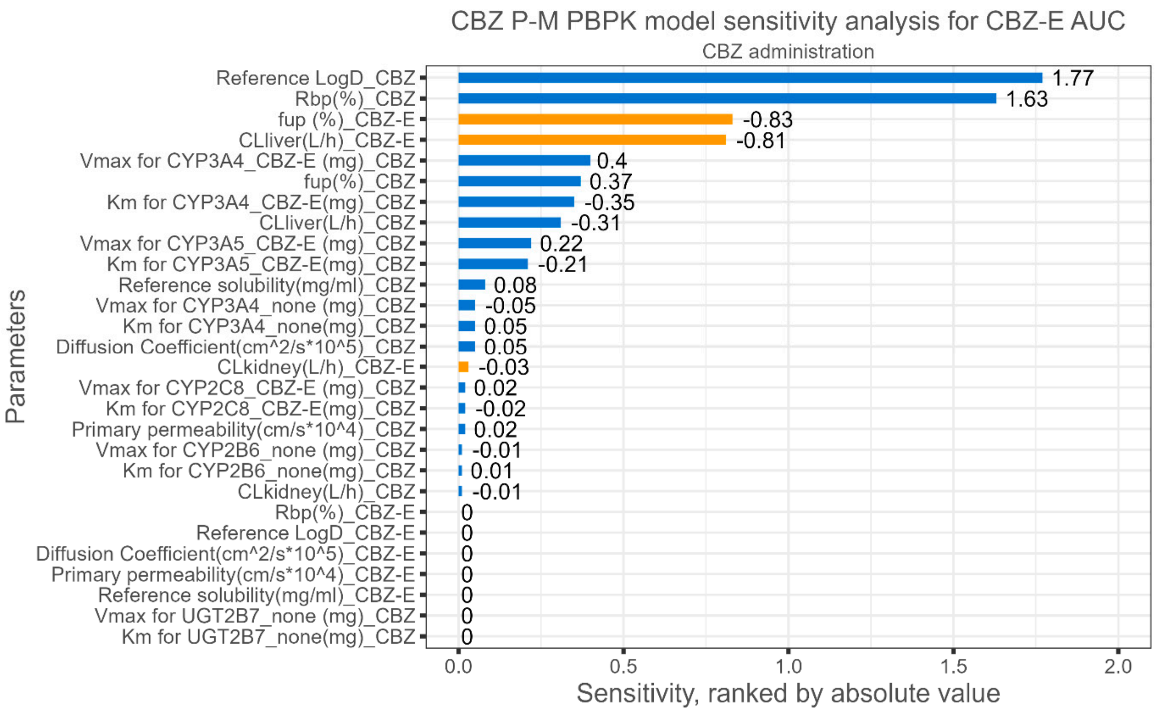

Figure S5 CBZ P-M PBPK model sensitivity analysis calculated as change of the simulated CBZ-E $AUC_{0\text{-inf}}$. Vmax: maximal reaction rate; km: Michaelis-Menten constant (half of the maximum reaction rate is achieved); CYP:cytochrome P450; UGT: UDP-glucuronosyltransferase; R_{bp}: blood to plasma ratio; fup: plasma unbound fraction; CL_{liver}: Liver clearance; CL_{kidney}: kidney clearance;

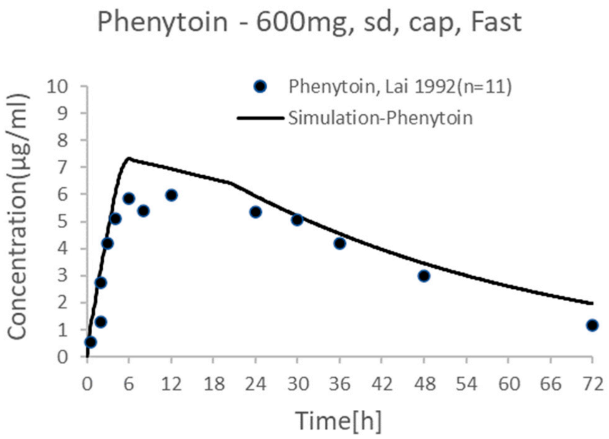

Figure S6 Comparing Simulated and Predicted Phase I Results of Phenytoin

(a)

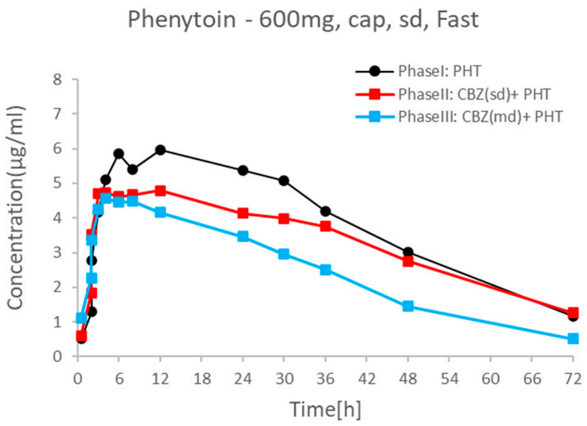

(b)

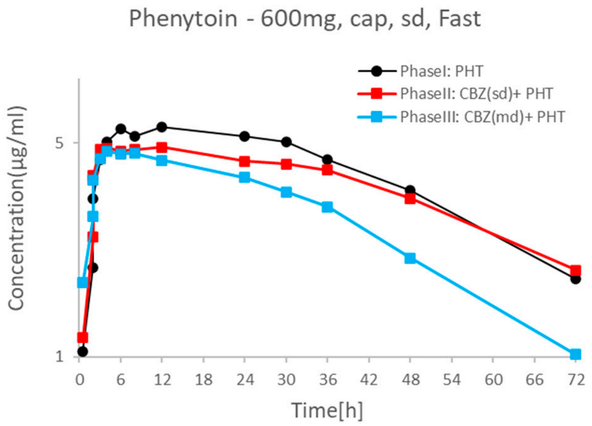

Figure S7 Comparative Analysis of Observed Phenytoin DDI Study Values

(a) Normal scale; (b) y-axis in log scale.

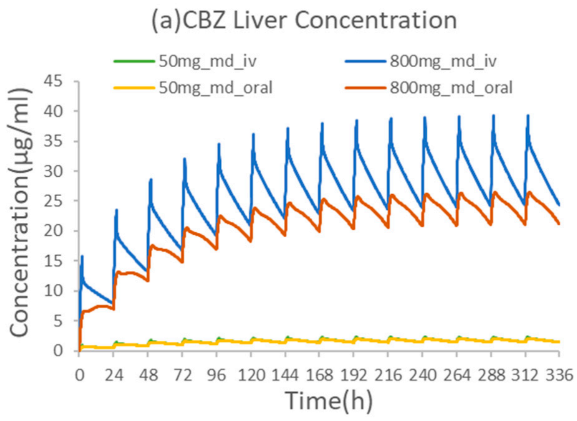

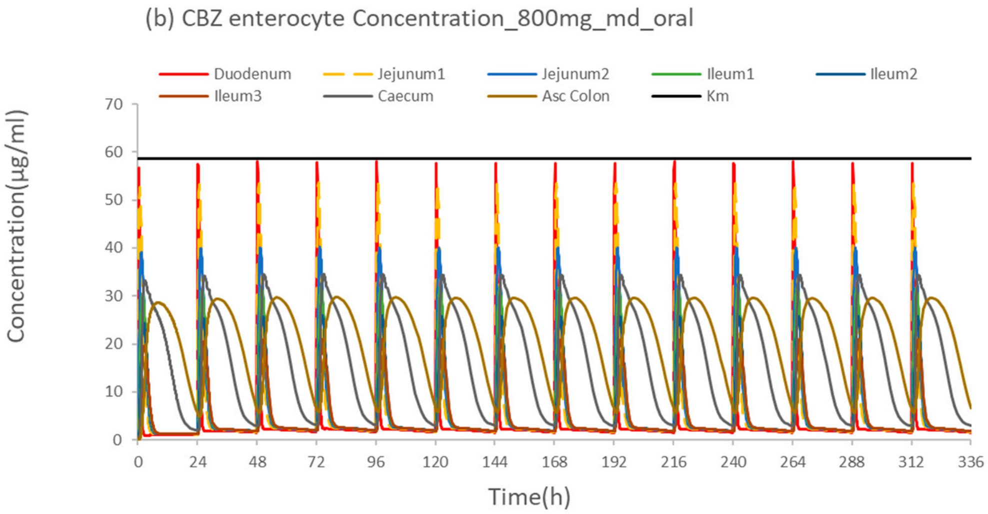

Figure S8 The clinical simulation results for CBZ nonlinear PK exploration

(a)Simulation result for CBZ Liver Concentrations; (b) Simulation result for CBZ Enterocyte Concentrations across different intestinal regions, when given 800mg IR tablet daily; the black horizontal line indicates 58.59 μg/ml; md: multiple dose; iv: iv infusion; oral: oral administration.

Reference

1. Tomson, T.; Tybring, G.; Bertilsson, L. Single-Dose Kinetics and Metabolism of Carbamazepine-10,11-Epoxide. *Clin Pharmacol Ther* **1983**, *33*, 58–65, doi:10.1038/clpt.1983.8.

2. Sumi, M.; Watari, N.; Umezawa, O.; Kaneniwa, N. Pharmacokinetic Study of Carbamazepine and Its Epoxide Metabolite in Humans. *J Pharmacobiodyn* **1987**, *10*, 652–661, doi:10.1248/bpb1978.10.652.

3. Pisani, F.; Caputo, M.; Fazio, A.; Oteri, G.; Russo, M.; Spina, E.; Perucca, E.; Bertilsson, L. Interaction of Carbamazepine-10,11-Epoxide, an Active Metabolite of Carbamazepine, with Valproate: A Pharmacokinetic Study. *Epilepsia* **1990**, *31*, 339–342, doi:10.1111/j.1528-1157.1990.tb05385.x.

4. Pisani, F.; Fazio, A.; Artesi, C.; Oteri, G.; Spina, E.; Tomson, T.; Perucca, E. Impairment of Carbamazepine-10, 11-Epoxide Elimination by Valnoctamide, a Valpromide Isomer, in Healthy Subjects. *Br J Clin Pharmacol* **1992**, *34*, 85–87, doi:10.1111/j.1365-2125.1992.tb04114.x.

5. Gérardin, A.; Dubois, J.P.; Moppert, J.; Geller, L. Absolute Bioavailability of Carbamazepine after Oral Administration of a 2% Syrup. *Epilepsia* **1990**, *31*, 334–338, doi:10.1111/j.1528-1157.1990.tb05384.x.

6. Rawlins, M.D.; Collste, P.; Bertilsson, L.; Palmér, L. Distribution and Elimination Kinetics of Carbamazepine in Man. *Eur J Clin Pharmacol* **1975**, *8*, 91–96, doi:10.1007/BF00561556.

7. Eichelbaum, M.; Toon, T.; Tybring, G.; Bertilsson, L. Carbamazepine Metabolism in Man: Induction and Pharmacogenetic Aspects. *Clin Pharmacokinet* **1985**, *10*, 80–90, doi:10.2165/00003088-198510010-00004.

8. Wada, J.A.; Troupin, A.S.; Friel, P.; Remick, R.; Leal, K.; Pearmain, J. Pharmacokinetic Comparison of Tablet and Suspension Dosage Forms of Carbamazepine. *Epilepsia* **1978**, *19*, 251–255, doi:10.1111/j.1528-1157.1978.tb04487.x.

9. Gérardin, A.P.; Abadie, F. v.; Campestrini, J.A.; Theobald, W. Pharmacokinetics of Carbamazepine in Normal Humans after Single and Repeated Oral Doses. *J Pharmacokinet Biopharm* **1976**, *4*, 521–535, doi:10.1007/BF01064556.

10. Bedada, S.K.; Nearati, P. Effect of Resveratrol on the Pharmacokinetics of Carbamazepine in Healthy Human Volunteers. *Phytother Res* **2015**, *29*, 701–706, doi:10.1002/ptr.5302.

11. Bedada, S.K.; Appani, R.; Boga, P.K. Effect of Piperine on the Metabolism and Pharmacokinetics of Carbamazepine in Healthy Volunteers. *Drug Res* **2017**, *67*, 46–51, doi:10.1055/s-0042-118173.

12. Kim, K.-A.; Oh, S.O.; Park, P.-W.; Park, J.-Y. Effect of Probenecid on the Pharmacokinetics of Carbamazepine in Healthy Subjects. *Eur J Clin Pharmacol* **2005**, *61*, 275–280, doi:10.1007/s00228-005-0940-7.

13. Meyer, M.C.; Straughn, A.B.; Jarvi, E.J.; Wood, G.C.; Pelsor, F.R.; Shah, V.P. The Bioinequivalence of Carbamazepine Tablets with a History of Clinical Failures. *Pharmaceutical Research: An Official Journal of the American Association of Pharmaceutical Scientists* **1992**, *9*, 1612–1616, doi:10.1023/A:1015872626887.

14. Meyer, M.C.; Straughn, A.B.; Mhatre, R.M.; Shah, V.P.; Williams, R.L.; Lesko, L.J. The Relative Bioavailability and in Vivo-in Vitro Correlations for Four Marketed Carbamazepine Tablets. *Pharm Res* **1998**, *15*, doi:10.1023/A:1011929300613.

15. Shahzadi, A.; Javed, I.; Aslam, B.; Muhammad, F.; Asi, M.R.; Ashraf, M.Y.; Zia-ur-Rahman Therapeutic Effects of Ciprofloxacin on the Pharmacokinetics of Carbamazepine in Healthy Adult Male Volunteers. *Pak J Pharm Sci* **2011**, *24*, 63–68.

16. Saint-Salvi, B.; Tremblay, D.; Surjus, A.; Lefebvre, M.A. A Study of the Interaction of Roxithromycin with Theophylline and Carbamazepine. *J Antimicrob Chemother* **1987**, *20 Suppl B*, 121–129, doi:10.1093/jac/20.suppl_b.121.

17. Barzaghi, N.; Gatti, G.; Crema, F.; Monteleone, M.; Amione, C.; Leone, L.; Perucca, E. Inhibition by Erythromycin of the Conversion of Carbamazepine to Its Active 10,11-Epoxide Metabolite. *Br J Clin Pharmacol* **1987**, *24*, 836–838, doi:10.1111/j.1365-2125.1987.tb03257.x.

18. Bianchetti, G.; Padovani, P.; Thénot, J.P.; Thiercelin, J.F.; Morselli, P.L. Pharmacokinetic Interactions of Progabide with Other Antiepileptic Drugs. *Epilepsia* **1987**, *28*, 68–73, doi:10.1111/j.1528-1157.1987.tb03625.x.

19. Faigle, J.W.; Feldmann, K.F. Pharmacokinetic Data of Carbamazepine and Its Major Metabolites in Man. In *Clinical Pharmacology of Anti-Epileptic Drugs*; Springer Berlin Heidelberg: Berlin, Heidelberg, 1975; pp. 159–165.

20. Kovaĉević, I.; Parojĉić, J.; Homŝek, I.; Tubić-Grozdanis, M.; Langguth, P. Justification of Biowaiver for Carbamazepine, a Low Soluble High Permeable Compound, in Solid Dosage Forms Based on IVIVC and Gastrointestinal Simulation. *Mol Pharm* **2009**, *6*, 40–47, doi:10.1021/mp800128y.

21. Graham, G.; Williams, K. Metabolism and Pharmacokinetics of Ibuprofen. In *Aspirin and Related Drugs*; CRC Press, 2004; Vol. 4, pp. 157–180 ISBN 9780203646960.

22. Pynnönen, S. The Pharmacokinetics of Carbamazepine in Plasma and Saliva of Man. *Acta Pharmacol Toxicol (Copenh)* **1977**, *41*, 465–471, doi:10.1111/j.1600-0773.1977.tb02157.x.

23. Wong, Y.Y.; Ludden, T.M.; Bell, R.D. Effect of Erythromycin on Carbamazepine Kinetics. *Clin Pharmacol Ther* **1983**, *33*, 460–464, doi:10.1038/clpt.1983.62.

24. Levy, R.H.; Pitlick, W.H.; Troupin, A.S.; Green, J.R.; Neal, J.M. Pharmacokinetics of Carbamazepine in Normal Man. *Clin Pharmacol Ther* **1975**, *17*, 657–668, doi:10.1002/cpt1975176657.

25. Dalton, M.J.; Powell, J.R.; Messenheimer, J.A. The Influence of Cimetidine on Single-Dose Carbamazepine Pharmacokinetics. *Epilepsia* **1985**, *26*, 127–130, doi:10.1111/j.1528-1157.1985.tb05395.x.

26. Dalton, M.J.; Powell, J.R.; Messenheimer, J.A. Ranitidine Does Not Alter Single-Dose Carbamazepine Pharmacokinetics in Healthy Adults. *Drug Intell Clin Pharm* **1985**, *19*, 941–944, doi:10.1177/106002808501901217.

27. Cotter, L.M.; Eadie, M.J.; Hooper, W.D.; Lander, C.M.; Smith, G.A.; Tyrer, J.H. The Pharmacokinetics of Carbamazepine. *Eur J Clin Pharmacol* **1977**, *12*, 451–456, doi:10.1007/BF00561065.

28. Graf, E. Bioäquivalenz; Qualitätsbewertung Wirkstoffgleicher Fertigarzneimittel. Hsg. von H. Blume Und E. Mutschler Unter Mitarb. von G. Wendt, G. Stenzhorn, M. Siewert Und M. Schäfer-Korting. 1. Erg.-Lfg. 1990, Govi-Verlag, Eschborn. Mit Ordner DM 118,–. *Pharm Unserer Zeit* **1990**, *19*, 223–223, doi:10.1002/pauz.19900190516.

29. Licht, D.; Zholkovsky, M.; Kaplan, R.; Friedman, M.; Yacobi, A.; Golander, Y.; Moros, D.; Levitt, B. European Patent Specification. Sustained Release Carbamazepine Formulation. EP 1044 681 B1 2005, *99*, 1–19.

30. Kovaĉević, I.; Parojĉić, J.; Homŝek, I.; Tubić-Grozdanis, M.; Langguth, P. Justification of Biowaiver for Carbamazepine, a Low Soluble High Permeable Compound, in Solid Dosage Forms Based on IVIVC and Gastrointestinal Simulation. *Mol Pharm* **2009**, *6*, 40–47, doi:10.1021/mp800128y.

31. Kshirsagar, R.; Shinde, G.; Kandikurwar, A. United States Patent Application Publication. Extended Release Pharmaceutical Compositions Containing Carbamazepine, Pub. No.: US 2014/0302138A1 2014, *1*.

32. Gande, M.; Gondalia, R.; Kothapalli, M.; Velishala, N.M.; Koppuri, V. United States Patent Application Publication - Carbamazepine Extended Release Dosage Form, Pub. No.: US 2009/01696.19 A1 2009, *1*, 1–3.

33. McLean, A.; Browne, S.; Zhang, Y.; Slaughter, E.; Halstenson, C.; Couch, R. The Influence of Food on the Bioavailability of a Twice-Daily Controlled Release Carbamazepine Formulation. *J Clin Pharmacol* **2001**, *41*, 183–186, doi:10.1177/00912700122010005.

34. Burstein, A.H.; Horton, R.L.; Dunn, T.; Alfaro, R.M.; Piscitelli, S.C.; Theodore, W. Lack of Effect of St John's Wort on Carbamazepine Pharmacokinetics in Healthy Volunteers. *Clin Pharmacol Ther* **2000**, *68*, 605–612, doi:10.1067/mcp.2000.111530.

35. Møller, S.E.; Larsen, F.; Khant, A.Z.; Rolan, P.E. Lack of Effect of Citalopram on the Steady-State Pharmacokinetics of Carbamazepine in Healthy Male Subjects. *J Clin Psychopharmacol* **2001**, *21*, 493–499, doi:10.1097/00004714-200110000-00007.

36. Ji, P.; Damle, B.; Xie, J.; Unger, S.E.; Grasela, D.M.; Kaul, S. Pharmacokinetic Interaction between Efavirenz and Carbamazepine after Multiple-Dose Administration in Healthy Subjects. *J Clin Pharmacol* **2008**, *48*, 948–956, doi:10.1177/0091270008319792.

37. Miles, M. V.; Tennison, M.B. Erythromycin Effects on Multiple-Dose Carbamazepine Kinetics. *Ther Drug Monit* **1989**, *11*, 47–52, doi:10.1097/00007691-198901000-00010.

38. Bernus, I.; Dickinson, R.G.; Hooper, W.D.; Eadie, M.J. Early Stage Autoinduction of Carbamazepine Metabolism in Humans. *Eur J Clin Pharmacol* **1994**, *47*, 355–360, doi:10.1007/BF00191168.

39. Andreasen, A.-H.; Brøsen, K.; Damkier, P. A Comparative Pharmacokinetic Study in Healthy Volunteers of the Effect of Carbamazepine and Oxcarbazepine on Cyp3a4. *Epilepsia* **2007**, *48*, 490–496, doi:10.1111/j.1528-1167.2007.00924.x.

40. Song, I.; Weller, S.; Patel, J.; Borland, J.; Wynne, B.; Choukour, M.; Jerva, F.; Piscitelli, S. Effect of Carbamazepine on Dolutegravir Pharmacokinetics and Dosing Recommendation. *Eur J Clin Pharmacol* **2016**, *72*, 665–670, doi:10.1007/s00228-016-2020-6.

41. Lai, M.L.; Lin, T.S.; Huang, J.D. Effect of Single- and Multiple-Dose Carbamazepine on the Pharmacokinetics of Diphenylhydantoin. *Eur J Clin Pharmacol* **1992**, *43*, 201–203, doi:10.1007/BF01740672.

42. Lutz, J.D.; Kirby, B.J.; Wang, L.; Song, Q.; Ling, J.; Massetto, B.; Worth, A.; Kearney, B.P.; Mathias, A. Cytochrome P450 3A Induction Predicts P-Glycoprotein Induction; Part 2: Prediction of Decreased Substrate Exposure After Rifabutin or Carbamazepine. *Clin Pharmacol Ther* **2018**, *104*, 1191–1198, doi:10.1002/cpt.1072.

43. Meyer, M.C.; Straughn, A.B.; Jarvi, E.J.; Wood, G.C.; Pelsor, F.R.; Shah, V.P. The Bioinequivalence of Carbamazepine Tablets with a History of Clinical Failures. *Pharm Res* **1992**, *9*, 1612–1616, doi:10.1023/a:1015872626887.

44. Meyer, M.C.; Straughn, A.B.; Mhatre, R.M.; Shah, V.P.; Williams, R.L.; Lesko, L.J. The Relative Bioavailability and in Vivo-in Vitro Correlations for Four Marketed Carbamazepine Tablets. *Pharm Res* **1998**, *15*, 1787–1791, doi:10.1023/a:1011929300613.

45. Kovacević, I.; Parojcić, J.; Homsek, I.; Tubić-Grozdanis, M.; Langguth, P. Justification of Biowaiver for Carbamazepine, a Low Soluble High Permeable Compound, in Solid Dosage Forms Based on IVIVC and Gastrointestinal Simulation. *Mol Pharm* **2009**, *6*, 40–47, doi:10.1021/mp800128y.

46. Morselli, P.L.; Gerna, M.; de Maio, D.; Zanda, G.; Viani, F.; Garattini, S. Pharmacokinetic Studies on Carbamazepine in Volunteers and in Epileptic Patients. In *Clinical Pharmacology of Anti-Epileptic Drugs*; Springer Berlin Heidelberg: Berlin, Heidelberg, 1975; pp. 166–180.

47. Shou, M.; Hayashi, M.; Pan, Y.; Xu, Y.; Morrissey, K.; Xu, L.; Skiles, G.L. Modeling, Prediction, and in Vitro in Vivo Correlation of CYP3A4 Induction. *Drug Metab Dispos* **2008**, *36*, 2355–2370, doi:10.1124/dmd.108.020602.

48. Moore, A.; Chothe, P.P.; Tsao, H.; Hariparsad, N. Evaluation of the Interplay between Uptake Transport and CYP3A4 Induction in Micropatterned Cocultured Hepatocytes. *Drug Metab Dispos* **2016**, *44*, 1910–1919, doi:10.1124/dmd.116.072660.

49. Savaryn, J.P.; Sun, J.; Ma, J.; Jenkins, G.J.; Stresser, D.M. Broad Application of CYP3A4 Liquid Chromatography-Mass Spectrometry Protein Quantification in Hepatocyte Cytochrome P450 Induction Assays Identifies Nonuniformity in MRNA and Protein Induction Responses. *Drug Metab Dispos* **2022**, *50*, 105–113, doi:10.1124/dmd.121.000638.

50. Zhang, J.G.; Patel, R.; Clark, R.J.; Ho, T.; Trisdale, S.K.; Fang, Y.; Stresser, D.M. Effect of Fifteen CYP3A4 in Vitro Inducers on the Induction of Hepatocytes : A Trend Analysis.Poster Presented at: 20th North American ISSX Meeting; 18-22 Oct; Orlando Florida.; 2015; p. 2015.

51. Vermet, H.; Raoust, N.; Ngo, R.; Esserméant, L.; Klieber, S.; Fabre, G.; Boulenc, X. Evaluation of Normalization Methods To Predict CYP3A4 Induction in Six Fully Characterized Cryopreserved Human Hepatocyte Preparations and HepaRG Cells. *Drug Metab Dispos* **2016**, *44*, 50–60, doi:10.1124/dmd.115.065581.

52. Sun, Y.; Chothe, P.P.; Sager, J.E.; Tsao, H.; Moore, A.; Laitinen, L.; Hariparsad, N. Quantitative Prediction of CYP3A4 Induction: Impact of Measured, Free, and Intracellular Perpetrator Concentrations from Human Hepatocyte Induction Studies on Drug-Drug Interaction Predictions. *Drug Metab Dispos* **2017**, *45*, 692–705, doi:10.1124/dmd.117.075481.

53. Fahmi, O.A.; Kish, M.; Boldt, S.; Obach, R.S. Cytochrome P450 3A4 MRNA Is a More Reliable Marker than CYP3A4 Activity for Detecting Pregnane X Receptor-Activated Induction of Drug-Metabolizing Enzymes. *Drug Metab Dispos* **2010**, *38*, 1605–1611, doi:10.1124/dmd.110.033126.

54. Kuramoto, S.; Kato, M.; Shindoh, H.; Kaneko, A.; Ishigai, M.; Miyauchi, S. Simple Evaluation Method for CYP3A4 Induction from Human Hepatocytes: The Relative Factor Approach with an Induction Detection Limit Concentration Based on the Emax Model. *Drug Metab Dispos* **2017**, *45*, 1139–1145, doi:10.1124/dmd.117.076349.

55. Zuo, R.; Li, F.; Parikh, S.; Cao, L.; Cooper, K.L.; Hong, Y.; Liu, J.; Faris, R.A.; Li, D.; Wang, H. Evaluation of a Novel Renewable Hepatic Cell Model for Prediction of Clinical CYP3A4 Induction Using a Correlation-Based Relative Induction Score Approach. *Drug Metabolism and Disposition* **2017**, *45*, 198–207, doi:10.1124/dmd.116.072124.

56. McGinnity, D.F.; Zhang, G.; Kenny, J.R.; Hamilton, G.A.; Otmani, S.; Stams, K.R.; Haney, S.; Brassil, P.; Stresser, D.M.; Riley, R.J. Evaluation of Multiple in Vitro Systems for Assessment of CYP3A4 Induction in Drug Discovery: Human Hepatocytes, Pregnane X Receptor Reporter Gene, and Fa2N-4 and HepaRG Cells. *Drug Metab Dispos* **2009**, *37*, 1259–1268, doi:10.1124/dmd.109.026526.
